# Implementation strategies to build mental health-care capacity in Malawi: a health-economic evaluation

**DOI:** 10.1016/S2214-109X(23)00597-1

**Published:** 2024-02-23

**Authors:** Juan Yanguela, Brian W Pence, Michael Udedi, Jonathan Chiwanda Banda, Kazione Kulisewa, Chifundo C Zimba, Jullita K Malava, Christopher Akiba, Josée M Dussault, Abigail M Morrison, Steve Mphonda, Mina C Hosseinipour, Bradley N Gaynes, Stephanie B Wheeler

**Affiliations:** Department of Health Policy and Management (J Yanguela MSc,Prof S B Wheeler PhD), Department of Epidemiology (Prof B W Pence PhD), and Department of Health Behavior (A M Morrison MPH), Gillings School of Global Public Health, University of North Carolina at Chapel Hill, Chapel Hill, NC, USA; Non-Communicable Diseases and Mental Health Division, Malawi Ministry of Health and Population, Department of Clinical Services, Lilongwe, Malawi (M Udedi PhD, J C Banda PhD); Malawi Epidemiology and Intervention Research Unit, Chilumba, Karonga District, Malawi (J K Malava MA); Department of Mental Health, Kamuzu University of Health Sciences, Blantyre, Malawi (K Kulisewa MMed); University of North Carolina Project-Malawi, Lilongwe, Malawi (C C Zimba PhD, S Mphonda BA, Prof M C Hosseinipour MD); RTI International, Research Triangle Park, NC, USA (C Akiba PhD); Department of Global Health, Institut Pasteur, Paris, France (J M Dussault PhD); Department of Medicine (Prof M C Hosseinipour) and Division of Global Mental Health, Department of Psychiatry (Prof B N Gaynes MD), University of North Carolina at Chapel Hill School of Medicine, Chapel Hill, NC, USA; Lineberger Comprehensive Cancer Center, University of North Carolina, Chapel Hill, NC, USA (Prof S B Wheeler)

## Abstract

**Background:**

Depression is a major contributor to morbidity and mortality in sub-Saharan Africa. Due to low system capacity, three in four patients with depression in sub-Saharan Africa go untreated. Despite this, little attention has been paid to the cost-effectiveness of implementation strategies to scale up evidence-based depression treatment in the region. In this study, we investigate the cost-effectiveness of two different implementation strategies to integrate the Friendship Bench approach and measurement-based care in non-communicable disease clinics in Malawi.

**Methods:**

The two implementation strategies tested in this study are part of a trial, in which ten clinics were randomly assigned (1:1) to a basic implementation package consisting of an internal coordinator acting as a champion (IC-only group) or to an enhanced package that complemented the basic package with quarterly external supervision, and audit and feedback of intervention delivery (IC + ES group). We included material costs, training costs, costs related to project-wide meetings, transportation and medication costs, time costs related to internal champion activities and depression screening or treatment, and costs of external supervision visits if applicable. Outcomes included the number of patients screened with the patient health questionnaire 2 (PHQ-2), cases of remitted depression at 3 and 12 months, and disability-adjusted life-years (DALYs) averted. We compared the cost-effectiveness of both packages to the status quo (ie, no intervention) using a micro-costing-informed decision-tree model.

**Findings:**

Relative to the status quo, IC + ES would be on average US$10 387 ($1349–$17 365) more expensive than IC-only but more effective in achieving remission and averting DALYs. The cost per additional remission would also be lower with IC + ES than IC-only at 3 months ($119 *vs* $223) and 12 months ($210 for IC + ES; IC-only dominated by the status quo at 12 months). Neither package would be cost-effective under the willingness-to-pay threshold of $65 per DALY averted currently used by the Malawian Ministry of Health. However, the IC + ES package would be cost-effective in relation to the commonly used threshold of three times per-capita gross domestic product per DALY averted.

**Interpretation:**

Investing in supporting champions might be an appropriate use of resources. Although not currently cost-effective by Malawian willingness-to-pay standards compared with the status quo, the IC + ES package would probably be a cost-effective way to build mental health-care capacity in resource-constrained settings in which decision makers use higher willingness-to-pay thresholds.

## Introduction

In low-income and middle-income countries (LMICs), depression is a top cause of disability and death.^1^ Despite this, many LMICs do not have specialised mental health services and suffer from an undersupply of mental health professionals;^2^ as such, these countries are unable to meet demand for mental health care.^3^ Indeed, in sub-Saharan Africa, more than three in four individuals with depression do not receive any treatment.^3^

Considering this provider undersupply, task-shifting is a promising strategy to improve access to mental health care in LMICs.^4^ Task-shifting involves upskilling primary care professionals and community members to integrate evidence-based care for common mental health conditions into existing care pathways.^4^ For example, the Friendship Bench intervention relies on training non-specialist health-care workers to deliver culturally appropriate, problem-solving therapy for depression.^5^ Furthermore, measurement-based care, which allows non-specialists to use validated depression symptom burden assessments to prescribe and adjust antidepressant treatment, has been shown to be safe, feasible, and efficacious in sub-Saharan Africa.^6,7^

The scale-up of task-shifting approaches in LMICs is hindered by uncertainty about the level of system investment necessary to ensure adequate fidelity and effectiveness outside of experimental conditions.^8,9^ Evidence on the cost-effectiveness of different implementation strategies is also lacking. In this study, we investigate the cost-effectiveness of two different implementation strategies to integrate the Friendship Bench approach and measurement-based care in non-communicable disease clinics in Malawi. Our specific objectives are to estimate the cost per patient screened, remission achieved, and disability-adjusted life-years (DALYs) averted under each of the two strategies. We expect that, using the status quo (ie, no intervention) as a reference, an enhanced implementation package combining champions with external supervision will be slightly costlier but more effective and cost-effective than a basic package including only champions.

## Methods

### Trial

#### Randomisation groups

The two implementation strategies tested in this study are part of the Sub-Saharan Africa Regional Partnership (SHARP) for Mental Health Capacity-Building Scale-Up trial (NCT03711786), a cluster-randomised trial done in Malawi. SHARP has been described in detail previously.^10^ In short, ten clinics were randomly assigned (1:1) to a basic implementation package consisting of an internal champion (IC-only group) or to an enhanced package that complemented the basic package with quarterly external supervision (IC + ES group). The same depression screening and treatment programme (described below) was implemented in both groups.

#### Training

In each clinic, three clinicians (nurses or clinical officers) were trained as internal champions and tasked with teaching providers at non-communicable disease clinics to administer the Patient Health Questionnaire 9 (PHQ-9) and to use the measurement-based care approach to manage antidepressant treatment. Furthermore, five or six patients per site were trained in the Friendship Bench intervention.

#### Clinical care

Upon presentation for non-communicable disease care, patients not already receiving depression treatment were given a mental health master card (serving as their medical chart) and were administered the PHQ-9 form. Providers were instructed to adhere to the following protocol: for patients who scored 0 on the first two items of the PHQ-9 (known as PHQ-2), no further action was recommended; patients who scored higher than 0 on the PHQ-2 were recommended to complete the remainder of the PHQ-9. PHQ-9 scores of less than 5 were deemed to require no further treatment; a depression consultation, including mental health education and mental health master card completion, was recommended for patients with a total PHQ-9 score of 5 or higher. Patients with scores between 5 and 9 and were recommended to receive the Friendship Bench intervention, whereas antidepressant treatment (1-month medication supply) was indicated for patients with scores of 10 or higher. Regardless of whether their score was higher or lower than 5, patients scoring higher than zero on question 9 of the PHQ-9 (which inquiries about suicidality) were expected to receive a suicidality assessment.

**Figure 1:**
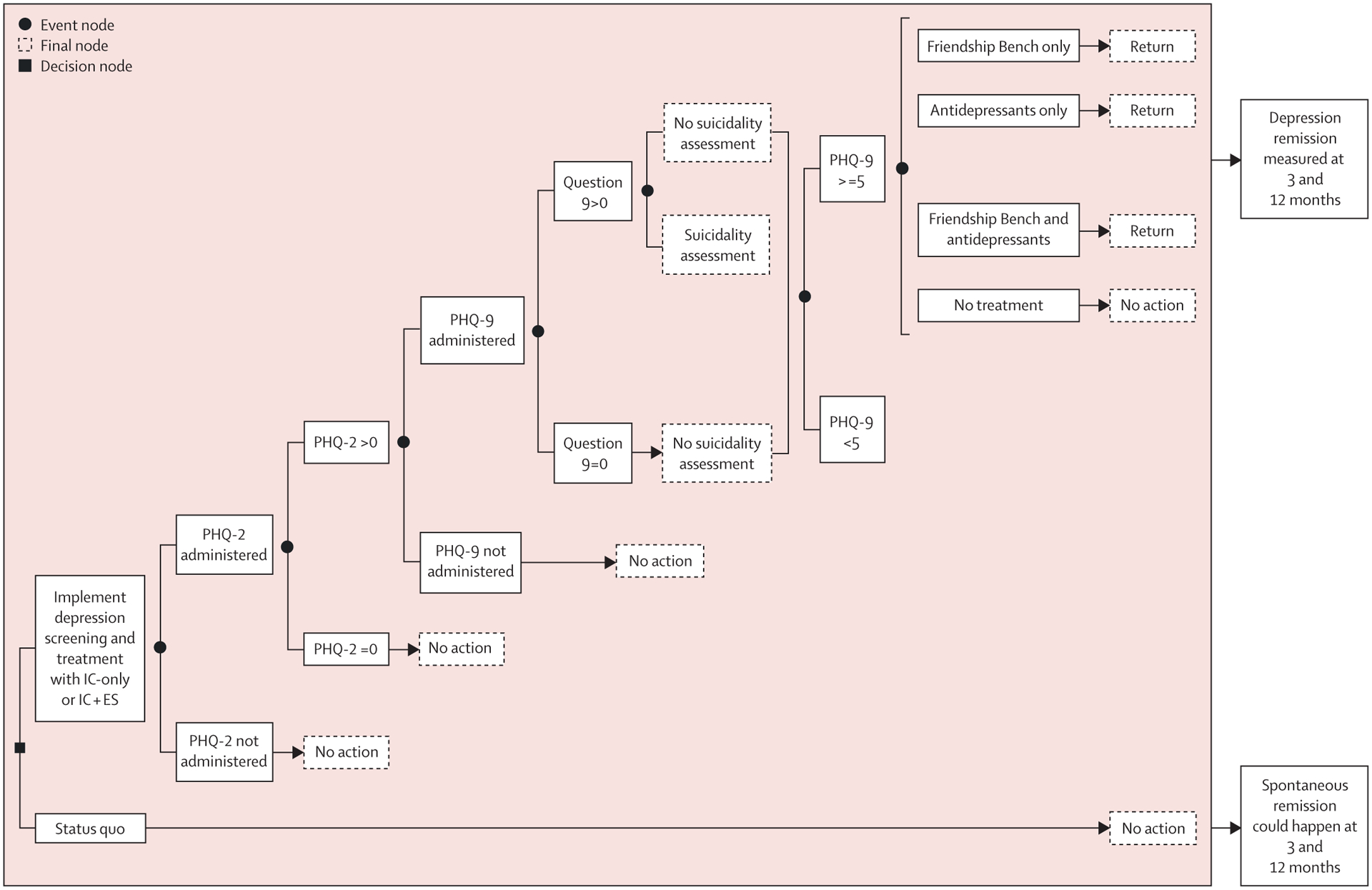
Clinical care flow for treatment-naive clinic visits The diagram describes the clinical care workflow for patients who have not previously started treatment for depression (ie, they represent new visits for treatment-naive patients with depression attending the non-communicable disease clinic and exclude return visits). IC-only represents the treatment package consisting of internal champions only, whereas IC + ES represents the treatment package consisting of internal champions plus external supervision. The decision node (depicted by a black square) represents the decision (ie, controllable factor) to implement depression screening and treatment using either implementation package (IC-only or IC + ES). Event nodes (black circles) indicate the presence of alternatives that can occur by chance (eg, the probability that a patient screens positive on the PHQ-2 or the probability that the PHQ-9 is administered after a positive PHQ-2, as per protocol). PHQ=Patient Health Questionnaire.

Although providers were encouraged to follow this treatment algorithm, they had discretion to prescribe the treatment they considered most appropriate. Additionally, in this pragmatic trial, there was considerable variability in the extent to which each step in this clinical flow process was followed (eg, some patients who scored positive on the PHQ-2 did not complete the PHQ-9 and some patients who scored positive on question 9 did not receive a suicidality assessment; figure 1). Previous analyses suggested that the implementation package (ie, IC-only or IC + ES) did not affect adherence to this algorithm, but had an effect on the proportion of patients achieving depression remission at 3 and 12 months (unpublished).

After initiating treatment, patients were re-assessed at their monthly follow-up visits at the non-communicable disease clinic, which involved reassessment with the PHQ-9 and any indicated continuation or adjustment to depression treatment.

#### Differences between packages

In the IC-only group, three clinicians per site were trained as internal champions (one principal coordinator and two alternates). Internal champions were responsible for record keeping, Friendship Bench supervision, ensuring antidepressant supply, raising implementation challenges with facility leadership, and preparing reports to the Ministry of Health. This basic implementation package is often used by the Ministry of Health to roll out new health programmes.

External supervision teams included two or three central government officials from the Ministry of Health, a Friendship Bench master trainer, and a person providing administrative and logistical support. External supervision visits focused on auditing paperwork, reviewing service delivery, and providing recommendations to the internal champion, the facility leadership, and the Ministry of Health.

#### Ethical approval

The SHARP for Mental Health Capacity-Building Scale-Up trial was approved by the University of North Carolina Biomedical Institutional Review Board (Chapel Hill, NC, USA; ID 250449) and the Malawian National Health Sciences Research Committee (Lilongwe, Malawi; #1925). No additional approval was needed for the cost-effectiveness analysis presented here.

### Perspective and context

We compared the cost-effectiveness of the two implementation strategies tested in the trial (which did not include an untreated group for ethical reasons) with the status quo (which assumes no treatment, as depression care is essentially absent from the health system due to inadequate infrastructure during the time of the trial). This comparison can help inform scale-up decisions about the relative benefits of implementing each strategy in facilities that currently offer no depression screening and no treatment, as is the case in most of Malawi and sub-Saharan Africa.

This analysis takes the perspective of the Ministry of Health scaling up each strategy to 14 of 28 districts in Malawi, including one set each of southern (n=5), central (n=5), and northern (n=4) region districts (appendix 1 p 1). These three sets of regional districts were selected because it would be logistically straightforward for an external supervision team based in Lilongwe (Malawi’s capital) to visit each set of districts in a single, approximately week-long trip per region. In Malawi, in terms of coverage, the current benefits package (which is provided free at the point of care to the entire population with no national insurance plan) covers depression screening and treatment (even if the infrastructure is not there to support it); as such, patients faced no out of pocket costs for visits or depression screening and treatment.

The selected set of 14 districts includes some, but not all, of the ten districts that participated in the SHARP trial, and includes some districts that did not take part. Observed trial parameters were applied to clinic volume data from the selected 14 districts to estimate the costs of a potential scale-up.

### Costs

We included material costs, training costs, costs related to project-wide meetings, transportation and medication costs, time costs related to internal champion activities and depression screening or treatment, and costs of external supervision visits if applicable (appendix 1 pp 1–14).

Costs were calculated in 2019 Malawian Kwacha (MWK) and 2019 US$. The exchange rate ($1=730 MWK) was stable throughout the trial (May 9, 2019–Nov 30, 2021). In appendix 1 (p 15), we report results in international dollars to facilitate comparison across economies that use different currencies and across which the prices of goods and services vary.

### Effectiveness

**Figure 2:**
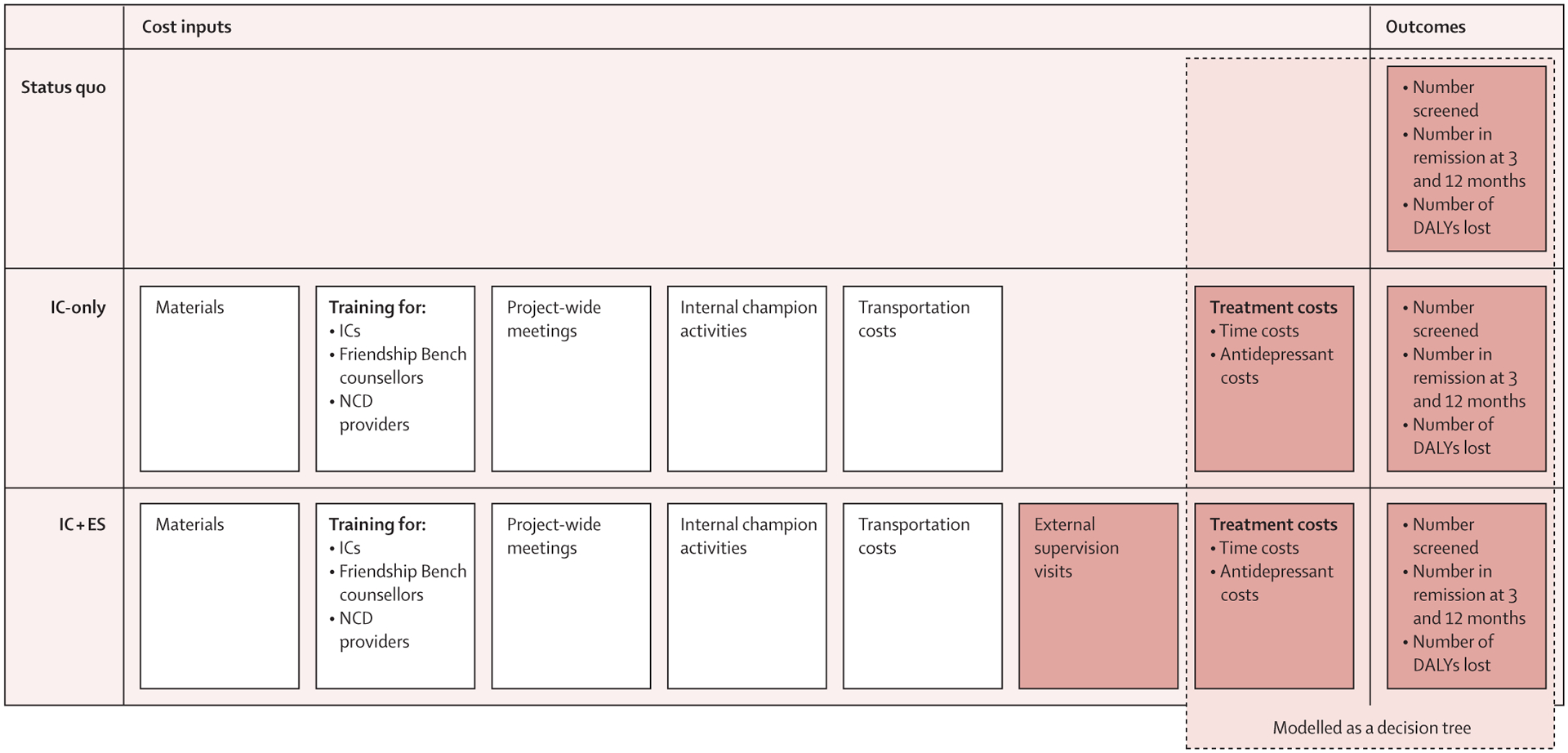
Cost inputs and outcomes by scenario The figure shows the cost and outcome-related inputs that were used for each of the scenarios modelled (ie, status quo, IC-only, and IC + ES). Material costs, training costs, costs related to project-wide meetings and internal champion activities, and transportation costs (as well as external supervision costs in the IC + ES alternative) were considered to be fixed costs (ie, cost associated with the scale-up to the selected 14 districts, independent of the number of patients seen). Treatment costs (which do depend on the number of patients seen) were calculated by summing the costs accrued by the cohort while moving through different alternatives of the clinical care decision tree depicted in figure 1. IC-only represents the treatment package consisting of internal champions only, whereas IC + ES represents the treatment package consisting of internal champions plus external supervision. Outcomes were also calculated using the clinical care decision tree depicted in figure 1. NCD=non-communicable disease. DALY=disability-adjusted life-year.

The following trial outcomes were included in the cost-effectiveness analysis: number of patients receiving the PHQ-2, cases of remitted depression at 3 and 12 months, and DALYs averted (figure 2). The proportion of patients screened with the PHQ-2 did not vary across groups (unpublished) and therefore was assumed to be the same for both packages. As part of the status quo, no patients received depression screening or treatment.

In the trial, the PHQ-9 was administered at baseline, 3 months, and 12 months. Patients were considered to screen positive for depression if they had scores of 5 or higher. Remission was defined as a score of less than 5 after a preceding positive screen.^11^ We were interested in the effectiveness of the implementation packages, and we therefore focused on the proportion of patients achieving remission in each group (ie, IC-only or IC + ES), regardless of the treatment they received.

We used published meta-analyses (derived from the international literature because of the absence of Malawi-specific data; appendix 1 pp 5–14) to estimate probabilities of spontaneous remission in the status-quo group, because not accounting for this would lead to an overestimation of DALYs averted under the IC-only and IC + ES approaches. These meta-analyses show that, without treatment, 17% of patients would be expected to achieve remission at 3 months and 53% would be expected to achieve remission at 12 months,^12–14^ compared with 55% for 3 months and 68% for 12 months under the IC + ES alternative. Under the IC-only alternative, patients would be expected to achieve a higher remission rate than patients without treatment at 3 months (36% *vs* 17%) but a slightly lower rate at 12 months (45% *vs* 53%). Because it was judged implausible for internal champions to lead to worse outcomes than the status quo, the remission probability for the status-quo group was set to be equal to that of the IC-only group at 12 months (ie, 45%).

DALYs were calculated using disability weights from the Global Burden of Disease study.^15^ Using trial-derived depression status at the beginning and end of each time interval, we calculated months lost to disability and converted them to DALYs. We accounted for depression severity at diagnosis—mild (PHQ-9 score 5–9), moderate (10–14), and severe (>14)—and, in the absence of data to suggest otherwise, we assumed that if remission did not occur, patients would stay at the same severity level throughout the year. We also assumed that if remission occurred, it happened halfway through the time interval.

### Model structure

We used a micro-costing-informed decision-tree model (figure 2). In our model, we calculated the additional fixed costs of the IC-only and IC + ES packages in relation to the status quo. These fixed costs included material costs, training costs, costs related to project-wide meetings and internal champion activities, and transportation costs (as well as external supervision costs in the IC + ES alternative). We then used a decision tree (figure 1) to calculate both effectiveness outcomes (number of patients receiving depression screening, remissions at 3 and 12 months, and DALYs averted), as well as variable costs (eg, personnel time, antidepressant cost) related to the flow of patients through the treatment algorithm.

Training requirements and start-up costs would be higher in the initial year of implementation (ie, when the packaged is introduced) compared with the second year, when the package is already in place (appendix 1 pp 1–4). Therefore, we report results separately for the first and second years.

The trial implemented the two packages (IC-only and IC + ES) and enrolled patients for a period of 30 months. However, we followed individual patients for 12 months after enrolment. The effectiveness of the IC + ES versus the IC-only packages in helping individual patients achieve remission at 3 and 12 months did not vary between patients enrolled during the first versus the second year of the intervention. Consequently, we assumed that effectiveness would remain constant in years 1 and 2. For both years, we used a cohort of 100 470 unique patients (ie, the number of patients who attended the 14 scale-up clinics in 2022). Patients were assumed to not have been screened for depression or have started treatment.

### Analyses

We calculated incremental cost-effectiveness ratios (ICERs) by dividing the difference in costs by the difference in each outcome in the IC + ES or IC-only groups compared with the status quo. To our knowledge, willingness-to-pay thresholds for an additional patient screened or in remission have not been defined globally. However, in LMICs, a threshold of three times per-capita gross domestic product (GDP) per DALY averted is considered cost-effective (whereas one time per-capita GDP is considered highly cost-effective).^16^ Nonetheless, governments across LMICs use different thresholds relevant to their economic contexts.^17^ For example, Malawi currently uses $65 per DALY averted as its willingness-to-pay threshold to inform decisions about the inclusion of interventions into its health benefit package.^18^ To make results more easily interpretable by a wider audience whose willingness-to-pay thresholds might change with time or with economic fluctuations, we present cost per DALY averted in relation to both one time ($584·4) and three times the 2019 Malawian per-capita GDP ($1753·2) as well as $65.

We also report cost-effectiveness acceptability curves, which indicate the probability that each alternative is cost-effective (ie, has a net monetary benefit) relative to the status quo under different willingness-to-pay thresholds. The net monetary benefit is calculated as incremental benefit multiplied by the willingness-to-pay threshold minus the incremental cost, with a positive net monetary benefit indicating that an alternative is cost-effective in relation to the status quo under that willingness-to-pay threshold.

We built the model in Excel (version 16.77.1) and used Crystal Ball (version 11.1.2.4.600; Oracle, Santa Clara, CA, USA) to conduct probabilistic sensitivity analyses across 1000 simulations drawn from individual parameter distributions (appendix 1 pp 5–14). We applied a 5% discount rate for costs and outcomes in year 2.^19^ We report average results across 1000 simulations and uncertainty bounds representing 95% of simulation runs.

### Role of the funding source

The funder of the study had no role in study design, data collection, data analysis, data interpretation, or writing of the report.

## Results

In year 1, IC + ES would have an average cost of $145 255 and IC-only would have an average cost of $134 867. As both alternatives would lead to the same average number of patients being screened (n=54 098), relative to the status quo, the ICER per patient screened would be lower (ie, more cost-effective) for IC-only than for IC + ES ($2·5 *vs* $2·7).

Compared with the status quo, IC + ES would lead to an average of 1268 additional remissions at 3 months, 772 additional remissions at 12 months, and 185 DALYs averted. By contrast, IC-only would result in an average of 624 additional remissions at 3 months, no additional remissions at 12 months, and 51 DALYs averted.

At 3 months, compared with the status quo, IC + ES would have a lower ICER per additional remission ($119) than IC-only ($223). For remission at 12 months, IC + ES would have an ICER of $210, whereas IC-only would be dominated by the status quo (ie, same number of remissions but higher costs). Regarding cost per DALY averted compared with status quo, IC + ES would have an average ICER of $812 and IC-only would have an average ICER of $2923 (table 1).

In year 2, (discounted) average total costs decrease from $134 867 to $86 530 for IC-only and $145 255 to $96 398 for IC + ES (table 2).

In year 2, relative to the status quo, IC-only would still be more cost-effective than IC + ES in terms of ICER per patient screened ($1·7 *vs* $1·9), whereas IC + ES would remain more cost-effective in terms of costs per remission at 3 months ($150 *vs* $83). IC-only would remain dominated by the status quo in relation to remission at 12 months.

Compared with the status quo, in year 2, IC + ES would have an ICER per DALY averted of $567, whereas IC-only would have an ICER per DALY averted of $1973 (table 2).

**Figure 3:**
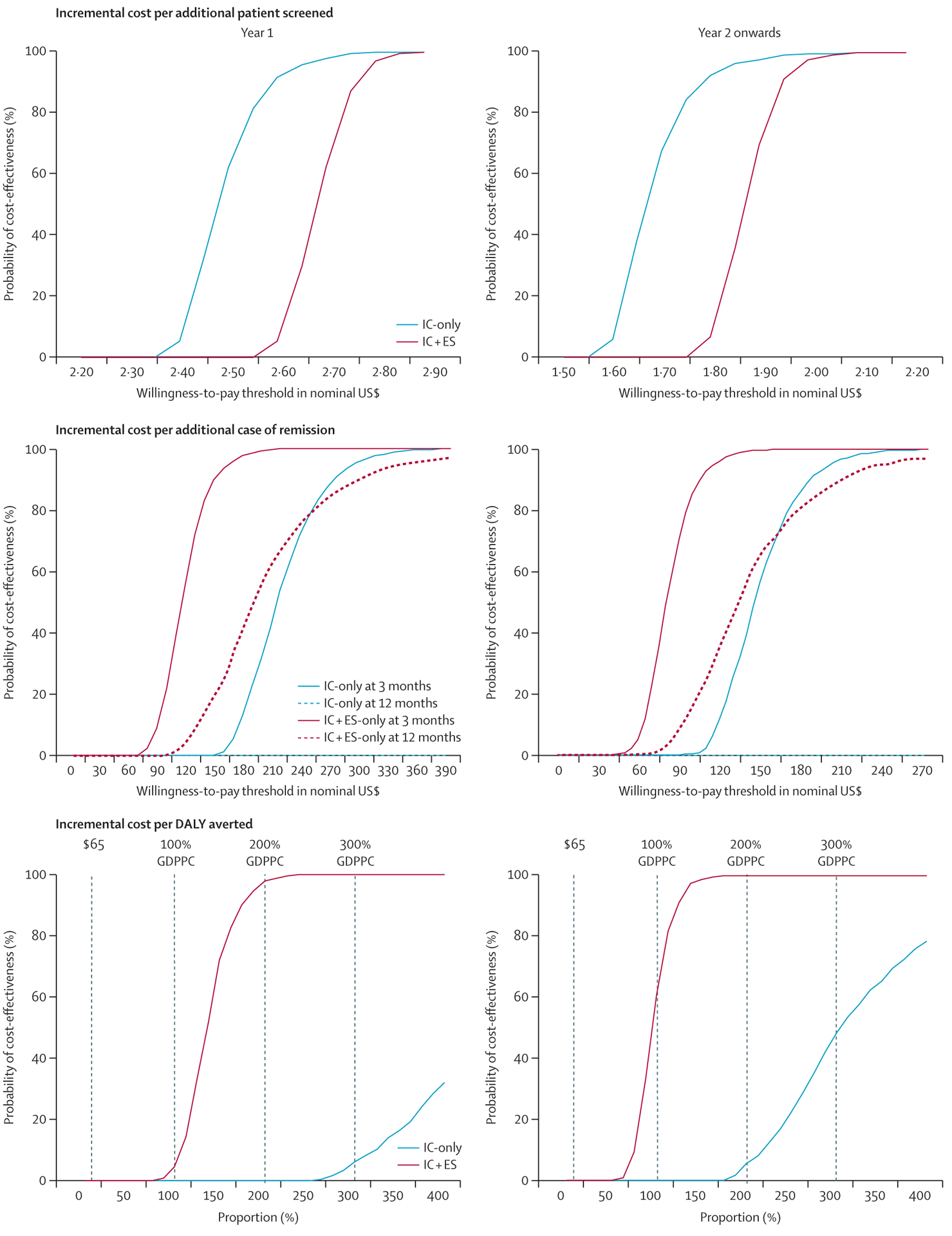
Cost-effectiveness acceptability curves for outcomes of interest Cost-effectiveness acceptability curves are constructed using the net monetary benefit approach. For each of the 1000 simulations, the net monetary benefit is calculated for a range of willingness-to-pay thresholds. Then, the percentage of simulations with a positive net monetary benefit under each threshold (ie, cost-effective in relation to each threshold) is calculated, allowing for a direct comparison of the cost-effectiveness of IC-only and IC + ES alternatives in relation to the status quo. IC-only represents the implementation package consisting of internal champions only, whereas IC + ES represents the implementation package consisting of internal champions plus external supervision. For the incremental costs per DALY averted, the vertical lines represent different willingness-to-pay thresholds: $65 (used by the Ministry of Health in Malawi) and one to three times per-capita GDP (which is commonly used in the LMIC cost-effectiveness literature). One time Malawian per-capita GDP in 2019 was $584·4, two times was $1168·8, and three times was $1753·2. GDP=gross domestic product. GDPPC=GDP per capita. LMIC=low-income and middle-income countries.

In year 1, both IC-only and IC + ES would be expected to be cost-effective (compared with the status quo) at least 90% of the time at willingness-to-pay thresholds of about $2·6 per patient screened for IC-only and $2·75 per patient screened for IC + ES (figure 3). In year 2, these willingness-to-pay thresholds would drop to around $1·80 for IC-only and $1·95 for IC + ES.

Relative to the status quo, in year 1, IC + ES would have a 90% probability of being cost-effective at willingness-to-pay thresholds of about $150 per additional remission at 3 months and $300 per additional remission at 12 months. By contrast, IC-only would only be expected to be cost-effective at least 90% of the time at a willingness-to-pay threshold of around US$280 per additional remission at 3 months (and it would be dominated by the status quo at 12 months). In year 2, the willingness-to-pay thresholds with a 90% probability of cost-effectiveness compared with the status quo would decrease to $105 per additional remission at 3 months and $210 per additional remission at 12 months for IC + ES, and to $190 per additional remission at 3 months for IC-only.

In terms of cost per DALY averted, in year 1, both implementation packages would have a 0% probability of being cost-effective relative to the status quo under a $65 willingness-to-pay threshold. More than 99% of IC + ES simulation results (but less than 10% of IC-only simulation trials) would be cost-effective under a willingness-to-pay threshold of three times per-capita GDP ($1753·2).

In year 2, both strategies would still have a 0% probability of being cost-effective compared with no treatment under the $65 willingness-to-pay threshold. However, around 60% of IC + ES simulations would be cost-effective under a willingness-to-pay threshold of one time per-capita GDP ($584·4). By contrast, less than 50% of IC-only simulations would be cost-effective compared with the status quo under the threshold of three times per-capita GDP ($1753·2). Appendix 1 (pp 16–19) includes ICER planes showing the individual results across 1000 simulation trials.

Cost-effectiveness frontiers allow for simultaneously comparing IC + ES, IC-only, and the status quo to each other (appendix 1 pp 20–24). They show the probability that each alternative has the highest net monetary benefit and is therefore the preferred alternative (ie, the most cost-effective) at different willingness-to-pay thresholds. The cost-effectiveness frontiers confirm the results of the cost-effectiveness acceptability curves. Finally, a secondary analysis directly comparing the cost-effectiveness of IC + ES with IC-only is included in appendix 1 (pp 25–30). Although this scenario would not be applicable to Malawi (as it assumes IC-only to be the standard of care), it is informative for decision makers evaluating whether to invest in external supervision in settings where internal champions are already in place.

## Discussion

Task-shifting approaches can successfully address common mental health conditions in LMICs.^7,20^ Accordingly, current research efforts focus on identifying implementation strategies to integrate mental health care into existing care pathways.^21^ Compared with the status quo, an enhanced implementation package including champions and external supervision would be more cost-effective than champions alone in terms of cost per remission at 3 and 12 months. Regarding cost per DALY averted, both packages are unlikely to reach cost-effectiveness in the current Malawian context, but the enhanced package would be cost-effective under a threshold of three times per-capita GDP. From year 2 onwards, it would also have a high probability (~60%) of being cost-effective at a threshold of one time per-capita GDP (figure 3).

Our findings align with the wider literature. Champions are an essential component of many implementation efforts.^22^ However, champion performance is affected by multiple factors, including the level of support from organisational leaders.^23^ It is likely that, in the IC + ES package, frequent interactions between internal champions, Ministry of Health representatives, and facility leadership created a more favourable implementation climate.^24^ Our own qualitative research^25^ highlighted the influence of the external supervision teams in helping internal champions overcome implementation barriers. In terms of the cost-effectiveness literature, we identified one study by Healey and colleagues assessing the cost-effectiveness of the Friendship Bench intervention in Zimbabwe.^26^ This study focused on the cost-effectiveness of the intervention itself (and not on the cost-effectiveness of implementation packages) and used efficacy data derived from experimental conditions (as opposed to effectiveness data from a pragmatic trial). It found that, compared with no intervention, the Friendship Bench intervention would have an ICER per DALY averted in the $191–$1823 range (depending on assumptions about the degree to which effectiveness and coverage would decrease outside of experimental conditions). These results are similar to the ICERs we found for IC + ES relative to the status quo. Our review also identified several protocols of trials that will assess the cost-effectiveness of different implementation strategies to scale-up task-shifting interventions for mental health disorders in sub-Saharan Africa.^27,28^

Our analysis is not without limitations. First, our cost-effectiveness estimates are conservative because they do not include remission-related productivity gains or productivity losses linked to untreated depression. In addition, we assumed a static population and, because our trial was randomised at the clinic level, we built a cohort-based model. This approach means that conclusions about cost-effectiveness are not relevant for individual patient trajectories, as our goal was to inform population-based policy. Finally, we were only able to follow patients for 12 months. Although a longer follow-up would have been desired, one of the major benefits of depression treatment is the acceleration of remission.^29^ Consequently, a time horizon of one year is informative and probably further contributes to the conservative nature of our estimates. Furthermore, although our model suggested substantially lower costs in year 2 (due to the fairly high budgetary impact of start-up training costs), it is important to note that costs might increase over time if staff turnover and subsequent training needs are high.

Our analyses also have numerous strengths. First, model inputs were derived from a large, clinic-randomised trial, which allowed us to obtain high-quality estimates of the impact of IC + ES and IC-only on implementation and patient outcomes. In addition, we assessed multiple outcomes, allowing for an exploration of how cost-effectiveness varies across time and endpoints. Furthermore, we used a comprehensive, activity-based costing strategy and drew upon local experts to design a scale-up approach that reduced costs (eg, decentralising training sessions, minimising travel). Moreover, our analyses are responsive to local decision-making needs. Up to this point, the Malawian Ministry of Health did not have access to DALY-based estimates of the cost-effectiveness of mental health-care programmes, making it hard to compare them with investments targeting other diseases. Finally, to our knowledge, this will be the first published cost-effectiveness analysis of implementation strategies to integrate mental health care into existing non-communicable disease care pathways in sub-Saharan Africa, a region that remains under-represented in the mental health implementation science literature.

To conclude, compared with the status quo, a pragmatic implementation strategy combining champions and external supervision would be only slightly more expensive but significantly more effective in achieving remission at the population level than an approach including only champions. Although currently this strategy is unlikely to be cost-effective in Malawi at the $65 willingness-to-pay threshold, it has a high probability of being cost-effective at a threshold of one to three times per-capita GDP. Therefore, it might be a crucial tool to build mental health-care capacity in other LMICs with higher willingness-to-pay thresholds. Our results should not be misinterpreted as evidence that the preferred option would be to leave depression untreated in Malawi. Rather, we simply report on the incremental costs and benefits that would be expected if the IC-only or the IC + ES packages were scaled up. It is possible that, in the future, IC + ES could become cost-effective if a change in economic conditions, health sector prioritisation, or mental health-focused donor programmes lead to an increase in the willingness-to-pay thresholds used to make health-care resource allocation decisions in Malawi. In the meantime, implementation research should aim to identify cost-effective strategies to implement and scale-up evidence-based interventions in the Malawian context or in settings using similar willingness-to-pay thresholds.

## Supplementary Material

1

2

## Figures and Tables

**Table 1:** Cost, outcomes, and ICERs for the IC-only and the IC+ES groups for year 1

	Status quo	IC-only	IC + ES
Screened patients	0	54 098 (53 887–54 322)	54 098 (53 887–54 322)
Patients with depression (expected)	3294 (3214–3366)	3294 (3214–3366)	3294 (3214–3366)
Patients in remission at 3 months	561 (435–684)	1184 (1073–1302)	1828 (1460–2240)
Patients in remission at 12 months	1483 (1354–1612)	1483 (1354–1612)	2255 (1854–2723)
Total depression-related DALYs	589 (513–665)	538 (470–604)	396 (330–459)
Total cost	0	$134 867 ($130 019-$142 557)	$145 255 ($140 712-$150 514)
Total cost, million MWK	0	98·5 (94·9–104·1)	106·0 (102·7–109·9)
Additional patients screened	Ref	54 098 (53 887–54 322)	54 098 (53 887–54 322)
Additional patients in remission at 3 months	Ref	624 (446–794)	1268 (884–1691)
Additional patients in remission at 12 months	Ref	0	772 (423–1199)
Additional DALYs averted	Ref	51 (27–77)	185 (132–245)
Additional cost	Ref	$134 867 ($130 019-$142 557)	$145 255 ($140 712-$150 514)
Additional cost, million MWK	Ref	98·5 (94·9–104·1)	106·0 (102·7–109·9)
ICER per additional patient screened	Ref	$2·5 ($2·4–$2·6)	$2·7 ($2·6–$2·8)
ICER per additional patient in remission at 3 months	Ref	$223 ($169-$299)	$119 ($86-$166)
ICER per additional patient in remission at 12 months	Ref	Dominated*	$210 ($121-$346)
ICER per additional DALY averted	Ref	$2923 ($1724-$5000)	$812 ($593-$1100)

We present average results across 1000 simulation trials and uncertainty bounds representing 95% of simulation runs. Costs are shown in nominal US$, unless otherwise stated. IC-only represents the implementation package consisting of internal champions only, whereas IC + ES represents the implementation package consisting of internal champions plus external supervision. DALY=disability-adjusted life-year. ICER=incremental cost-effectiveness ratio. MWK=Malawian Kwacha.

* In terms of incremental cost per additional remission at 12 months, the IC-only strategy would be dominated by the status quo (ie, in our model, it would lead to the same number of remissions but at a higher cost).

**Table 2:** ICERs for the IC-only and the IC + ES alternatives for year 2

	ICER per additional patient screened	ICER per additional patient in remission at 3 months	ICER per additional patient in remission at 12 months	ICER per additional DALY averted	Total discounted cost, million MWK	Total discounted cost
Status quo	Ref	Ref	Ref	Ref	Ref	Ref
IC-only	$1·7 ($1·6–$1·8)	$150 ($114-$202)	Dominated*	$1973 ($1158-$3401)	63·2 (60·0–68·5)	$86 530 ($82 198-$93 813)
IC + ES	$1·9 ($1·8–$2·0)	$83 ($60-$115)	$147 ($85-$243)	$567 ($414-$772)	70·4 (67·3–73·9)	$96 398 ($92 259-$101 270)

We present average results across 1000 simulation trials and uncertainty bounds representing 95% of simulation runs. Costs are shown in nominal US$, unless otherwise stated. IC-only represents the implementation package consisting of internal champions only, whereas IC + ES represents the implementation package consisting of internal champions plus external supervision. DALY=disability-adjusted life-year. ICER=incremental cost-effectiveness ratio. MWK=Malawian Kwacha.

* In terms of incremental cost per additional remission at 12 months, the IC-only strategy would be dominated by the status quo (ie, in our model, it would lead to the same number of remissions but at a higher cost).

## Data Availability

Individual patient-level data collected as part of the study will be made publicly available to all researchers (for any research purposes) after publication of the Article through the National Institutes of Mental Health Data Archive. Data available through the National Institute of Mental Health Data Archive will include individual de-identified participant data and the data dictionary. The study protocol and the analytical plan will be available (after publication of the Article) upon request. Requests can be addressed to Dr Brian W Pence (bpence@unc.edu).

## Research in context

### Evidence before this study

Depression is one of the main drivers of mortality and morbidity in low-income and middle-income countries. However, insufficient system infrastructure for mental health care in these settings results in most individuals with depression not receiving treatment. Trials have shown the feasibility and efficacy of non-specialist-led depression care in low-resource settings. Yet scale-up of these evidence-based interventions is often hindered by uncertainty about the level of system investment necessary to ensure adequate fidelity and effectiveness outside of experimental conditions. Evidence on the cost-effectiveness of different implementation strategies is also lacking. On Nov 27, 2023, we systematically searched PubMed and EMBASE (without restrictions on publication date or language of publication) for literature evaluating the cost-effectiveness of different strategies to implement task-shifting interventions to treat common mental health conditions in sub-Saharan Africa. We used the following search terms: “cost-effectiveness” AND (“task-shifting” OR “task-sharing”) AND (“mental health” OR “depression”) AND “sub-Saharan Africa”. Our search did not retrieve any relevant health-economic evaluations but identified several protocols of trials that will evaluate the comparative cost-effectiveness of different implementation packages to scale up task-shifted mental health-care models in the region.

### Added value of this study

This study compares the cost-effectiveness of two common facility-level implementation strategies—internal champions and external audit and feedback—relative to the status quo of no depression screening and no treatment, to integrate non-specialist-led depression care into existing pathways for chronic care conditions. Internal champions alone would achieve adequate screening rates, accelerate remission among patients diagnosed with depression, and avert life-years lost to disability. However, despite being only marginally more expensive, a combined implementation approach, combining internal champions and external supervision, would further accelerate remission and avert a larger number of years lost to disability than using internal champions alone. Compared with the status quo, none of the implementation packages would be cost-effective under the willingness-to-pay threshold currently used in Malawi, where the study was done. Nonetheless, the combined implementation strategy would have a high probability of being cost-effective under a threshold of one to three times the per-capita gross domestic product per disability-adjusted life-year averted, which is often used in resource-constrained settings.

### Implications of all the available evidence

These findings suggest that a combined implementation strategy, including both internal champions and external supervision, might provide a crucial tool in scaling up integration of evidence-based mental health treatment into general medical care in low-income and middle-income settings at a compelling cost-effectiveness level.
