# Supplementary material for: Implementation strategies to build mental health-care capacity in Malawi: a health-economic evaluation
Source: Lancet Glob Health. Author manuscript; Available in PMC 2024 Apr 1. (PMC10958395; doi:10.1016/S2214-109X(23)00597-1)
Supplement: 1 [file NIHMS1976842-supplement-1.pdf]

THE LANCET
Global Health

Supplementary appendix 1

This appendix formed part of the original submission and has been peer reviewed. We post it as supplied by the authors.

Supplement to: Yanguela J, Pence BW, Udedi M, et al. Implementation strategies to build mental health-care capacity in Malawi: a health-economic evaluation. *Lancet Glob Health* 2024; published online Feb 23. https://doi.org/10.1016/S2214-109X(23)00597-1.

SUPPLEMENTARY MATERIALS

Appendix 1. List of sites for a potential scale up

Central region sites

- Nkhotakhota
- Salima
- Ntchisi
- Dowa
- Kasungu

Southern region sites

- Mangochi
- Balaka
- Machinga
- Mulanje
- Phalombe

Northern Region

- Mzimba
- Nkhata Bay
- Karonga
- Chilumba

Appendix 2. List of cost-inputs included in the model

- **Material costs:** pens, pads (for trainees and trainers), markers and flipcharts (for trainers).
- **Training costs:**
    - ***Training for Implementation Champions (ICs)***
        - *Initial IC training:*
            - Purpose: to train ICs.
            - Location and frequency: one central meeting held in Lilongwe (LLW) before the launch of the intervention.
            - Trainees: three ICs from each of the 14 sites (one principal IC and two alternates) travel to LLW by bus.
            - Number of trainers: four trainers based in LLW.
            - Number of administrative support staff: two (based in LLW).
            - Duration: three days.
        - *Supplemental IC training:*
            - Purpose: to train ICs.
            - Location and frequency: one central meeting held in Lilongwe (LLW) every year, starting a year after the launch of the intervention.

- Trainees: two ICs from each of the 14 sites (one principal IC and one alternate) travel to LLW by bus.
- Number of trainers: four trainers based in LLW.
- Number of administrative support staff: two (based in LLW).
- Duration: two days.

- ***Training for Non-Communicable Diseases (NCD) providers***
  - *Initial NCD training*
    - Purpose: to train NCD providers.
    - Location and frequency: one training session held in each of the 14 sites before the launch of the intervention.
    - Trainees: 20 NCD providers employed at the site.
    - Number of trainers: three. The three ICs at the site act as trainers.
    - Number of administrative support staff: one.
    - Duration: two days.

  - *Supplemental NCD training*
    - Purpose: to train NCD providers.
    - Location and frequency: one training session held in each of the 14 sites every year, starting a year after the launch of the intervention.
    - Trainees: 20 NCD providers employed at the site
    - Number of trainers: three. The three ICs at the site act as trainers.
    - Number of administrative support staff: one.
    - Duration: one day.

- **Training for FB counselors**
  - *Initial training for FB counselors*
    - Purpose: to train NCD providers
    - Location and frequency: three training sessions, one in LLW for the central region sites, one in Zomba for the southern region sites, and one in Mzuzu for the northern region sites. All held before the launch of the intervention.
    - Trainees: five or six FB counselors per site. They travel to LLW, Zomba, or Mzuzu by bus.
    - Number of trainers: three per training session. They are based in LLW, and they travel to Zomba and Mzuzu by car.
    - Number of administrative support staff: one per training session. They are based in LLW, and they travel to Zomba and Mzuzu by car.
    - Duration: 12 days.

        - *Additional training for FB counselors (refresher training for previously trained counselors and training for new counsellors recruited to buffer attrition).*
            - Purpose: to train NCD providers.
            - Location and frequency: three training sessions, one in LLW for the central region sites, one in Zomba for the southern region sites, and one in Mzuzu for the northern region sites. All held before the launch of the intervention.
            - Trainees: All previously trained counselors (refresher training) and new counselors recruited to buffer for attrition (attrition training). Based on trial data, we assumed that there would be around 20% turnover in counselor turnover. FB counselors travel to LLW, Zomba, or Mzuzu by bus.
            - Number of trainers: 2 per training session. They are based in LLW, and they travel to Zomba and Mzuzu by car.
            - Number of administrative support staff: 1 per training session. They are based in LLW, and they travel to Zomba and Mzuzu by car.
            - Duration: seven days for newly recruited counselors and two days for previously trained ones.
    - **ES visits (specific to the IC+ES arm)**
        - Purpose: to audit paperwork, review service delivery (including ABCD and FB), and provide recommendations to the IC, the facility leadership, and the MoH.
        - Location and frequency: three visits per site per year.
        - Number of ES members: three members. We assumed that the ES members would be based in LLW and that they would divide up ES visits by region, traveling by car.
            - They would spend one week visiting sites in the Northern Region:
                - Sunday: Travel LLW-Mzimba.
                - Monday: Visit Mzimba. Travel Mzimba-Nkhata Bay.
                - Tuesday: Visit Nkhata Bay.
                - Wednesday: Travel Nkhata Bay-Karonga.
                - Thursday: Visit Karonga.
                - Friday: Travel Karonga-Chilumba. Visit Chilumba.
                - Saturday: Travel Chilumba-LLW.
            - They would spend one week visiting sites in the Southern Region:
                - Sunday: Travel LLW-Mangochi.
                - Monday: Visit Mangochi. Travel Mangochi-Balaka.
                - Tuesday: Visit Balaka. Travel Balaka-Machinga.
                - Wednesday: Visit Machinga. Travel Machinga-Mulanje
                - Thursday: Visit Mulanje. Travel Mulanje-Phalombe.
                - Friday: Visit Phalombe.
                - Saturday: Travel Phalombe-LLW.
            - They would spend one week visiting sites in the Central Region:
                - Sunday: Travel Lilongwe-Nkhotakhota.

            - Monday: Visit Nkhotakhota. Travel Nkhotakhota-Salima.
            - Tuesday: Visit Salima. Travel Salima-Ntchisi.
            - Wednesday: Visit Ntchisi. Travel Ntchisi-Dowa.
            - Thursday: Visit Dowa. Travel Dowa-Kasungu.
            - Friday: Visit Kasungu. Travel Kasungu-Lilongwe.
        - Duration: one day at each site.
    - **FB supervision (specific to the IC-only arm)**
        - Purpose: in the IC-only arm, FB trainers visit FB at each facility to provide feedback on FB activities specifically (without meeting with leadership or completing any of the other activities that are part of ES visits).
        - Location and frequency: three visits per site per year.
        - Supervisor: one regional FB trainer visits the sites in that region, traveling back and forth on the day by car.
            - We assume that the regional trainers for the central, northern, and southern region would be based in LLW, Mzuzu, and Zomba, respectively.
        - Duration: one day at each site.
    - **Project-wide meetings**
        - Purpose: to gather all clinics participating in the intervention to discuss successes and challenges
        - Location and frequency: semiannual meetings in LLW.
        - Attendees: one District Health Official (DHO)/ one District Medical Official (DMO)/ and two clinical staff per site. DHOs and DMOs travel by car, while the other staff travel by bus.
        - Duration: one day.

Appendix 3. Full list of model inputs

Cost inputs for training events, External Supervision (ES) visits, and project wide-meetings.

Input	Base case	Distributional assumption for PSA	Reference
Facilitation allowance for trainers	MWK 10,000	Fixed	Trial budget
Per diem (daily subsistence allowance)	MWK 30,000	Fixed	Trial budget
Lunch allowance	MWK 4,000	Fixed	Trial budget
Breakfast	MWK 4,000	Fixed	Trial budget
Refreshments	MWK 3,550	Triangular<br>Min:3300<br>Max: 3,800<br>Likeliest: 3,550	Trial budget
Materials and supplies for trainees	MWK 725	Triangular<br>Min: 600<br>Max: 850<br>Likeliest: 725	Trial budget
Materials and supplies for trainers	MWK 6,527	Triangular<br>Min:5,553<br>Max: 7,500<br>Likeliest: 6,527	Trial budget
Allowance for admin person	MWK 4,000	Fixed	Trial budget
Location rental	MWK 35,000	Triangular<br>Min:10,000<br>Max: 35,000<br>Likeliest: 60,000	Trial budget

Cost inputs associated to depression screening and treatment.

Input	Base case	Distributional assumption for PSA	Reference
Cost inputs common to both IC-only and ES alternatives			
Proportion of new patients that are screened with PHQ2	0.538 (44,728/83,068)	Beta (calculated using a three-point estimation technique with a likeliest value of 0.538, and 5% and 95% confidence bounds of 0.535 and 0.542, respectively).	Trial data
Proportion of PHQ2 that are positive	0.168 (7,500/44,728)	Beta (calculated using a three-point estimation technique with a likeliest value of 0.168, and 5% and 95% confidence bounds of 0.164 and 0.171, respectively).	Trial data
Proportion of PHQ2 positive patients that are screened with PHQ9	0.890 (6,674/7,500)	Beta (calculated using a three-point estimation technique with a likeliest value of 0.890, and 5% and 95% confidence bounds of 0.882 and 0.897, respectively).	Trial data

Proportion of patients screened with PHQ9 that screen positive for suicidality (Q9)	0.193 (1,286/6,674)	Beta (calculated using a three-point estimation technique with a likeliest value of 0.193, and 5% and 95% confidence bounds of 0.183 and 0.202, respectively).	Trial data
Proportion of patients who screen positive for suicidality (Q9) who receive suicidality assessment	0.936 (1,204/1,286)	Beta (calculated using a three-point estimation technique with a likeliest value of 0.936, and 5% and 95% confidence bounds of 0.923 and 0.950, respectively).	Trial data
Proportion of patients who screen positive for depression (score >=5) in the PHQ9	0.408 (2,722/6,674)	Beta (calculated using a three-point estimation technique with a likeliest value of 0.408, and 5% and 95% confidence bounds of 0.396 and 0.420, respectively).	Trial data
Proportion of patients with depression referred to FB (and not prescribed antidepressants)	0.802 (689/859)	Modeled as a Dirichlet distribution constructed by linking the following beta distributions and constraining their sum to one. Beta distributions were calculated using a three-point estimation technique based on the likeliest value and 95 confidence bounds.<br><br>Proportion prescribed FB:<br>0.802 (0.773-0.827)<br><br>Proportion prescribed AD:<br>0.144 (0.121-0.168)<br><br>Proportion prescribed both FB and AD:<br>0.048 (0.033-0.062)<br><br>Proportion prescribed neither:<br>0.006 (0.000-0.011)	Trial data
Proportion of patients with depression prescribed AD (and not referred to FB)	0.144 (124/859)		Trial data
Proportion of patients with depression prescribed both FB and AD	0.048 (41/859)		Trial data
Proportion of patients with depression prescribed neither FB nor AD	0.006 (5/859)		Trial data
Duration of AD treatment	3 months	Fixed	Assumption
Average of FB visits	2.81 sessions	Fixed	Trial data
FB counsellor hourly wage	MWK 900/h	Fixed	Trial data
NCD nurse hourly wage	MWK 1,373/h	Triangular<br>Min:1,260<br>Max: 1,487<br>Likeliest: 1,373	University of North Carolina – Malawi project team

Clinical Officer/ Implementation Champion hourly wage	MWK 2,267/h	Triangular<br>Min:2,043<br>Max: 2,491<br>Likeliest: 2,267	University of North Carolina – Malawi project team
Cost of antidepressant medication (one month supply)	MWK 284	Triangular:<br>Min: Amitriptylin (MWK 160)<br>Max: Fluoxetine (MWK 408)<br>Likeliest: average	University of North Carolina – Malawi project team
Mastercard	MWK 55	Fixed	University of North Carolina – Malawi project team
MWK to $USD conversion rate	MWK730 = $1	Fixed	Assumption based on conversion rate during trial
Proportion of patients with return visits	0.067 (5971/89039)	Beta (calculated using a three-point estimation technique with a likeliest value of 0.067, and 5% and 95% confidence bounds of 0.066 and 0.068 respectively).	Trial data
Time cost inputs applicable to the IC package only			
Administration of the PHQ2- NEW visits	1 min	Beta (calculated using a three-point estimation technique with a likeliest value of 1 , and 5% and 95% confidence bounds of 0.9 and 1.1, respectively).	Trial data
Depression consultation and treatment WITH suicidality assessment- NEW visits	7 min	Triangular<br><br>Min: 5<br>Max: 8<br>Likeliest: 7	Trial data
Depression consultation and treatment WITHOUT suicidality assessment- NEW visits	3 min	Triangular<br><br>Min: 2<br>Max: 4<br>Likeliest: 3	Trial data
FB counselling session- NEW visits	40 min	Triangular<br><br>Min: 31<br>Max: 46<br>Likeliest: 40	Trial data
AD prescription- NEW visits	1 min	Beta (calculated using a three-point estimation technique with a likeliest value of 1 , and 5% and 95% confidence bounds of 0.9 and 1.1, respectively).	Trial data
Administration of the PHQ2- RETURN visits	1 min	Beta (calculated using a three-point estimation technique with a likeliest value of 1 , and 5% and 95% confidence bounds of 0.9 and 1.1, respectively).	Trial data

Depression consultation and treatment WITH suicidality assessment- RETURN visits	6 min	Triangular<br><br>Min: 5<br>Max: 9.5<br>Likeliest: 6	Trial data
Depression consultation and treatment WITHOUT suicidality assessment- RETURN visits	3 min	Triangular<br><br>Min: 3<br>Max: 4<br>Likeliest: 3	Trial data
FB counselling session- RETURN visits	28 min	Triangular<br><br>Min: 19<br>Max: 356<br>Likeliest: 28	Trial data
Time spent by ICs in clinical meetings, clinical reporting, and additional coordinator duties	9h/month	Negative binomial	Trial data
Cost inputs applicable to the ES+IC package only			
Administration of the PHQ2- NEW visits	1 min	Beta (calculated using a three-point estimation technique with a likeliest value of 1 , and 5% and 95% confidence bounds of 0.9 and 1.1, respectively).	Trial data
Depression consultation and treatment WITH suicidality assessment- NEW visits	7 min	Triangular<br><br>Min: 5<br>Max: 8<br>Likeliest: 7	Trial data
Depression consultation and treatment WITHOUT suicidality assessment- NEW visits	3 min	Triangular<br><br>Min: 2<br>Max: 5<br>Likeliest: 3	Trial data
FB counselling session- NEW visits	35	Triangular<br><br>Min: 30<br>Max: 48<br>Likeliest: 35	Trial data
AD prescription- NEW visits	2 min	Beta (calculated using a three-point estimation technique with a likeliest value of 1 , and 5% and 95% confidence bounds of 0.9 and 1.1, respectively).	Trial data
Administration of the PHQ2- RETURN visits	1 min	Beta (calculated using a three-point estimation technique with a likeliest value of 1 , and 5% and 95% confidence bounds of 0.9 and 1.1, respectively).	Trial data
Depression consultation and treatment WITH	10 min	Triangular<br><br>Min: 6	Trial data

suicidality assessment- RETURN visits		Max: 21<br>Likeliest: 10	
Depression consultation and treatment WITHOUT suicidality assessment- RETURN visits	5 min	Triangular<br><br>Min: 4<br>Max: 6<br>Likeliest: 5	Trial data
FB counselling session- RETURN visits	22	Triangular<br><br>Min: 15<br>Max: 32<br>Likeliest: 22	Trial data
Time spent by ICs in clinical meetings, clinical reporting, and additional coordinator duties	16h/ month	Negative binomial	Trial data

Abbreviations: Internal Coordinators (ICs); External Supervision (ES); Friendship Bench (FB); Antidepressant (AD); Patient Health Questionnaire (PHQ); Malawian Kwacha (MWK); US Dollar (USD). Notes. Depression screening and consultation was handled evenly by nurses and clinical officers (each type of provider seeing 50% of patients). However, during the trial, some clinical teams adopted a different approach in which the PHQ2 was administered by nurses, who, if the PHQ2 was positive, would look for a clinical officer to administer the PHQ9. This variation of the general workflow was incorporated into the cost-effectiveness models (1). In both the IC and the ES+IC scenarios, the duration of FB sessions was multiplied by two to account for preparation and paperwork related activities (2). Our model accounts for the cost of training all three Champions (the main one and the two alternates). Consequently, although Champion attrition is not modeled directly, our model provides a conservative estimate that accounts for the costs of training alternate Champions that could step in in case of potential attrition (3).

<u>Inputs related to health outcomes</u>

Input	Base case	Distributional assumption for PSA	Reference
Proportion of mild cases among identified patients with depression	0.717 (477/665)	Modeled as a Dirichlet distribution constructed by linking the following beta distributions and constraining their sum to one. Beta distributions were calculated using a three-point estimation technique based on the likeliest value and 95 confidence bounds.<br><br>Proportion of mild cases: 0.717 (0.683-0.752)<br><br>Proportion of moderate severity cases: 0.128 (0.102-0.153)<br><br>Proportion of severe cases: 0.155 (0.127-0.182)	Trial data
Proportion of moderate cases among identified patients with depression	0.128 (85/665)		Trial data
Proportion of severe cases among identified patients with depression	0.155 (103/665)		Trial data
Probability of spontaneous depression remission at 3 months (without treatment)	0.173	Beta (calculated using a three-point estimation technique with a likeliest value of 0.17, and 5% and 95% confidence bounds of 0.114 and 0.23, respectively).	Whiteford *et al.* (2013), Mekonen *et al.* (2021), and Mekonen *et al.* (2022) (14–16)
Probability of spontaneous depression remission at 12 months (without treatment)	0.451 (107/237)	Beta (calculated using a three-point estimation technique with a likeliest value of 0.451, and 5% and 95% confidence bounds of 0.387 and 0.513, respectively).	Assumed to be the same as in the IC arm.
Probability of depression remission at 3 months in the IC arm	0.360 (99/275)	Beta(calculated using a three-point estimation technique with a likeliest value of 0.360, and 5% and 95% confidence bounds of 0.303 and 0.417, respectively).	Trial data
Probability of depression remission at 12 months in the IC arm	0.451 (107/237)	Beta (calculated using a three-point estimation technique with a likeliest value of 0.451, and 5% and 95% confidence bounds of 0.387 and 0.513, respectively).	Trial data

Risk ratio of depression remission at 3 months IC+ES vs IC only	1.526 ((145/264)/(99/275))	Lognormal Ln(mean)=0.422, Ln (standard error)=0.098	Trial data
Risk ratio of depression remission at 12 months IC+ES vs IC only	1.504 ((146/215)/(107/237))	Lognormal Ln(mean)=0.408, Ln (standard error)=0.086	Trial data
Disutility value for mild depression	0.145	Beta (calculated using a three-point estimation technique with a likeliest value of 0.15, and 5% and 95% confidence bounds of 0.099 and 0.209, respectively).	Global Burden of Disease (GBD) Mental Disorder Collaborators (17)
Disutility value for moderate depression	0.396	Beta (calculated using a three-point estimation technique with a likeliest value of 0.396, and 5% and 95% confidence bounds of 0.267 and 0.531, respectively).	Global Burden of Disease (GBD) Mental Disorder Collaborators (17)
Disutility value for severe depression	0.658	Beta (calculated using a three-point estimation technique with a likeliest value of 0.658, and 5% and 95% confidence bounds of 0.477 and 0.807, respectively).	Global Burden of Disease (GBD) Mental Disorder Collaborators (17)

Abbreviations: Internal Coordinators (ICs); External Supervision (ES); Low- and Middle-Income Countries (LMIC); Sub-Saharan Africa (SSA); Disability-adjusted Life Year (DALY). Notes: To account for the rates of spontaneous remission in the absence of treatment, which was not measured directly in our trial, we used estimates from the peer-reviewed literature. One meta-analysis by Whiteford at al., 2013 (15) estimated that spontaneous remission at 3 and 12 months was 23 and 53%, respectively. This analysis included data from waitlist and primary care samples, therefore overrepresenting mild and moderate cases of major depressive disorder, similar to the patients included in our trials. Another, more recent meta-analysis by Mekonen et al., 2022 (16) gave an overall pooled remission rate of 12.5% at 3 months. In this meta-analysis, data for the analyses were drawn from waitlist controls, so they did not have severe depression (i.e., they had mild-moderate depression, consistent with our sample). A third meta-analysis by Mekonen et al., 2021 (14) found that 11.4% of patients with depression achieved spontaneous depression without treatment within three months. To account for the variability observed in these meta-analyses, we assumed that spontaneous remission without treatment at 3 months would be modelled as a beta distribution centered around 17% (the average of 23% and 11.4%). The IC only arm achieved a higher remission rate than expected with no treatment at 3 months (36% vs 17%), but a slightly lower rate at 12 months (45%). Since it was judged implausible for the IC arm to lead to worse outcomes than the status quo, the remission probability for the status quo alternative was set to be equal to that of the IC only alternative at 12 months (i.e., 45%). Although these meta-analytic estimates of spontaneous remission are derived from community samples with predominantly mild cases (similar to our trial) and from a wide range of countries, the vast majority are drawn from samples in high-income countries. Both Whiteford at al., 2013 (15) and Mekonen et al., 2021 (14) only included estimates from high income countries. However, Mekonen et al., 2022 (16) included 2 studies from middle-income countries that did not significantly differ from the overall results. While we acknowledge that the use of Malawi-specific data would have been preferred, in the absence of SSA or LMIC-specific estimates, we consider that including an estimate of the probability of achieving remission in the absence of treatment is essential to avoid overestimating DALYs averted under the IC-only or IC+ES alternatives.

Transportation costs

Input	Base case	Distributional assumption for PSA	Source
Bus from Nkhotakhota to LLW	MWK 14,000	Fixed	University of North Carolina – Malawi project team
Bus from Salima to LLW	MWK 6,000	Fixed	University of North Carolina – Malawi project team
Bus from Ntchisi to LLW	MWK 6,000	Fixed	University of North Carolina – Malawi project team
Bus from Dowa to LLW	MWK 4,000	Fixed	University of North Carolina – Malawi project team
Bus from Kasungu to LLW	MWK 6,000	Triangular<br>Min: 6,000 (minibus)<br>Max: 12,000 (coach)<br>Likeliest: 6,000	University of North Carolina – Malawi project team
Bus from Mangochi to LLW	MWK 12,000	Fixed	University of North Carolina – Malawi project team
Bus from Balaka to LLW	MWK 13,000	Fixed	University of North Carolina – Malawi project team
Bus from Machinga to LLW	MWK 15,000	Fixed	University of North Carolina – Malawi project team
Bus from Mulanje to LLW	MWK 26,000	Fixed	University of North Carolina – Malawi project team
Bus from Phalombe to LLW	MWK 25,000	Fixed	University of North Carolina – Malawi project team
Bus from Mzimba to LLW	MWK 17,000	Fixed	University of North Carolina – Malawi project team
Bus from Nkhata Bay to LLW	MWK 24,500	Fixed	University of North Carolina – Malawi project team
Bus from Karonga to LLW	MWK 25,000	Fixed	University of North Carolina – Malawi project team
Bus from Chilumba to LLW	MWK 25,000	Fixed	University of North Carolina – Malawi project team
Daily cost of commuting for local attendees	MWK 2,000	Triangular.<br>Min: 1,000<br>Max: 4,000<br>Likeliest: 2,000	Trial budgets
Bus from Mangochi to Zomba	MWK 8,000.00	Fixed	University of North Carolina – Malawi project team
Bus from Balaka to Zomba	MWK 8,000.00	Fixed	University of North Carolina – Malawi project team

Bus from Machinga to Zomba	MWK 4,000.00	Fixed	University of North Carolina – Malawi project team
Bus from Mulanje to Zomba	MWK 9,000.00	Fixed	University of North Carolina – Malawi project team
Bus from Phalombe to Zomba	MWK 4,000.00	Fixed	University of North Carolina – Malawi project team
Bus from Mzimba to Mzuzu	MWK 12,000.00	Fixed	University of North Carolina – Malawi project team
Bus from Nkhata Bay to Mzuzu	MWK 4,500.00	Fixed	University of North Carolina – Malawi project team
Bus from Karonga to Mzuzu	MWK 11,000.00	Fixed	University of North Carolina – Malawi project team
Bus from Chilumba to Mzuzu	MWK 10,000.00	Fixed	University of North Carolina – Malawi project team
Distance from Zomba to LLW	288 km	Fixed	Google maps
Distance from Mzuzu to LLW	355 km	Fixed	Google maps
Distance from Lilongwe to Nkhotakhota.	172 km	Fixed	Google maps
Distance from Nkhotakhota to Salima.	112 km	Fixed	Google maps
Distance from Salima to Ntchisi.	109 km	Fixed	Google maps
Distance from Ntchisi to Dowa.	42 km	Fixed	Google maps
Distance from Dowa to Kasungu.	117 km	Fixed	Google maps
Distance from Kasungu to Lilongwe.	125 km	Fixed	Google maps
Distance from LLW to Mangochi.	247 km	Fixed	Google maps
Distance from Mangochi to Balaka.	77 km	Fixed	Google maps
Distance from Balaka to Machinga.	50 km	Fixed	Google maps
Distance from Machinga to Mulanje	136 km	Fixed	Google maps
Distance from Mulanje to Phalombe.	44 km	Fixed	Google maps
Distance from Phalombe to LLW.	357 km	Fixed	Google maps
Distance from LLW to Mzimba	272 km	Fixed	Google maps
Distance from Mzimba to Nkhata Bay.	160 km	Fixed	Google maps
Distance from Nkhata Bay to Karonga.	231 km	Fixed	Google maps
Distance from Karonga to Chilumba.	72 km	Fixed	Google maps
Distance from Chilumba to LLW.	508 km	Fixed	Google maps

Distance from Nkhotakhota to LLW	172 km	Fixed	Google maps
Distance from Salima to LLW	98.4 km	Fixed	Google maps
Distance from Ntchisi to LLW	89.4 km	Fixed	Google maps
Distance from Dowa to LLW	48.8 km	Fixed	Google maps
Distance from Kasungu to LLW	125 km	Fixed	Google maps
Distance from Mangochi to LLW	247 km	Fixed	Google maps
Distance from Balaka to LLW	206 km	Fixed	Google maps
Distance from Machinga to LLW	256 km	Fixed	Google maps
Distance from Mulanje to LLW	378 km	Fixed	Google maps
Distance from Phalombe to LLW	357 km	Fixed	Google maps
Distance from Mzimba to LLW	272 km	Fixed	Google maps
Distance from Nkhata Bay to LLW	387 km	Fixed	Google maps
Distance from Karonga to LLW	573 km	Fixed	Google maps
Distance from Chilumba to LLW	508 km	Fixed	Google maps
Distance from Mangochi to Zomba	246 km	Fixed	Google maps
Distance from Balaka to Zomba	164.4 km	Fixed	Google maps
Distance from Machinga to Zomba	68.8 km	Fixed	Google maps
Distance from Mulanje to Zomba	218 km	Fixed	Google maps
Distance from Phalombe to Zomba	152.2 km	Fixed	Google maps
Distance from Mzimba to Mzuzu	212 km	Fixed	Google maps
Distance from Nkhata Bay to Mzuzu	98.4 km	Fixed	Google maps
Distance from Karonga to Mzuzu	436 km	Fixed	Google maps
Distance from Chilumba to Mzuzu	308 km	Fixed	Google maps
L/100km	15	Fixed	Trial budget
Fuel cost (USD$/L)	1.34	Fixed	Trial budget

Abbreviations: Lilongwe (LLW); US dollar (USD); Liter (L); Malawian Kwacha (MWK).

Appendix 4. Results in international dollars

According to the World Bank, an international dollar is an artificial currency that "would buy in the cited country a comparable amount of goods and services a U.S. dollar would buy in the United States". https://datahelpdesk.worldbank.org/knowledgebase/articles/114944-what-is-an-international-dollar

Therefore, the use of international dollars makes it possible to compare purchasing power between economies that use different currencies and across which the prices of goods and services vary. In other words, International Dollars adjust for Purchasing Power Parity (PPP)

By contrast, nominal (or actual) US dollars, represent the actual value of a US dollar in a given year, without considering differences in purchasing value across settings.

Considering this, in this appendix, we present our results denominated in international dollars.

Year 1

	Additional # patients screened	*Additional # patients in remission at 3 months*	*Additional # patients in remission at 12 months*	*Additional DALYs averted*	*Additional cost (in international dollars)*
Status quo	Reference	Reference	Reference	Reference	
IC only	54,098 (53,887 – 54,322)	624 (446 – 794)	0	51 (27 – 77)	357,460 (344,609 – 377,841)
IC+ES	54,098 (53,887 – 54,322)	1,268 (884 – 1,691)	772 (423 – 1,199)	185 (132 – 245)	384,991(372,952– 398,932)
	ICER per one additional patient screened (in international dollars)	ICER per one additional patient in remission at 3 months (in international dollars)	ICER per one additional patient in remission at 12 months (in international dollars)	ICER per one additional DALY averted (in international dollars)	
Status quo	Reference	Reference	Reference	Reference	
IC only	6.6 (6.4–7.0)	590 (449–792)	Dominated*	7,747 (4,569–13,252)	
IC+ES	7.1 (6.9–7.4)	316 (227–441)	557 (321–917)	2,151 (1,573–2,916)	

Year 2

	ICER per one additional patient screened (in international dollars)	*ICER per one additional patient in remission at 3 months (in international dollars)*	*ICER per one additional patient in remission at 12 months (in international dollars)*	*ICER per one additional DALY averted (in international dollars)*
Status quo	Reference	Reference	Reference	Reference
IC only	4.5 (4.2–4.8)	398 (302–534)	Dominated*	5,231 (3,069–9,014)
IC+ES	5.0 (4.8–5.2)	221 (159–306)	389 (224–643)	1,503 (1,097–2,045)

International dollars were calculated by dividing 2019 Malawian Kwacha by the 2019 Purchasing Power Parity conversion factor (275.42) provided by the World Bank. (19)

<u>Appendix 5. Individual simulation results.</u>

Year 1

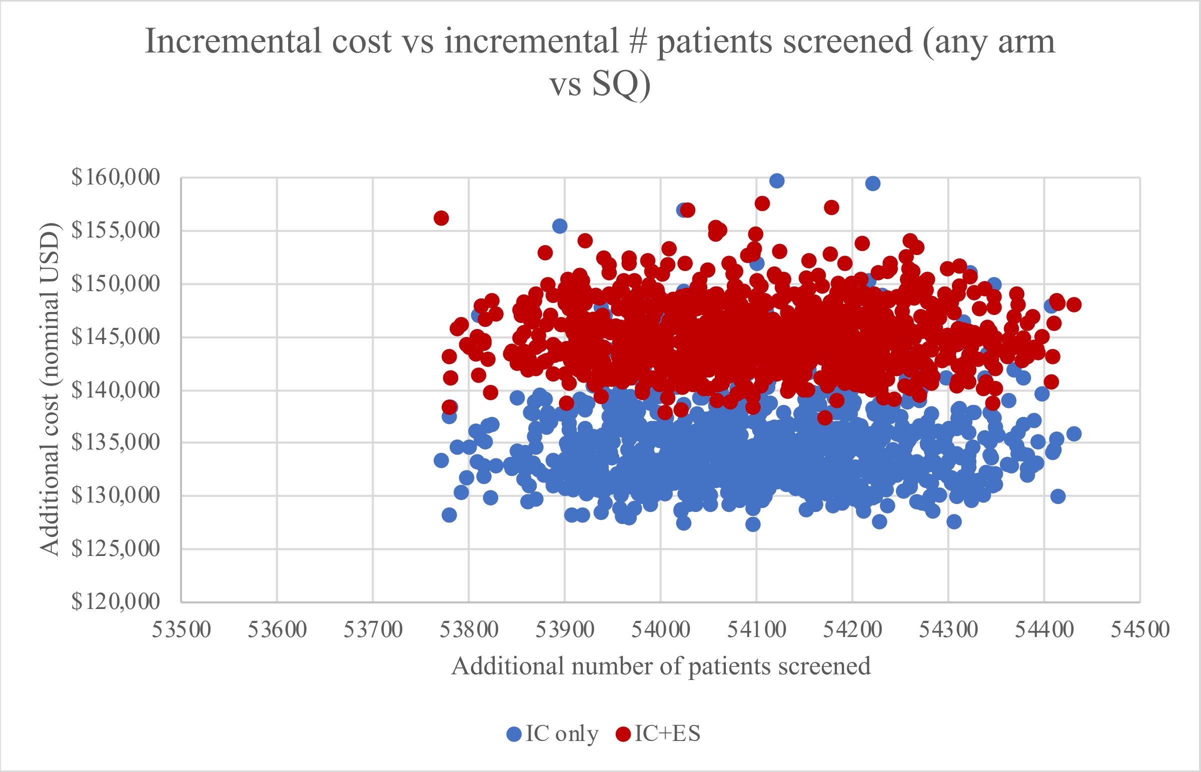

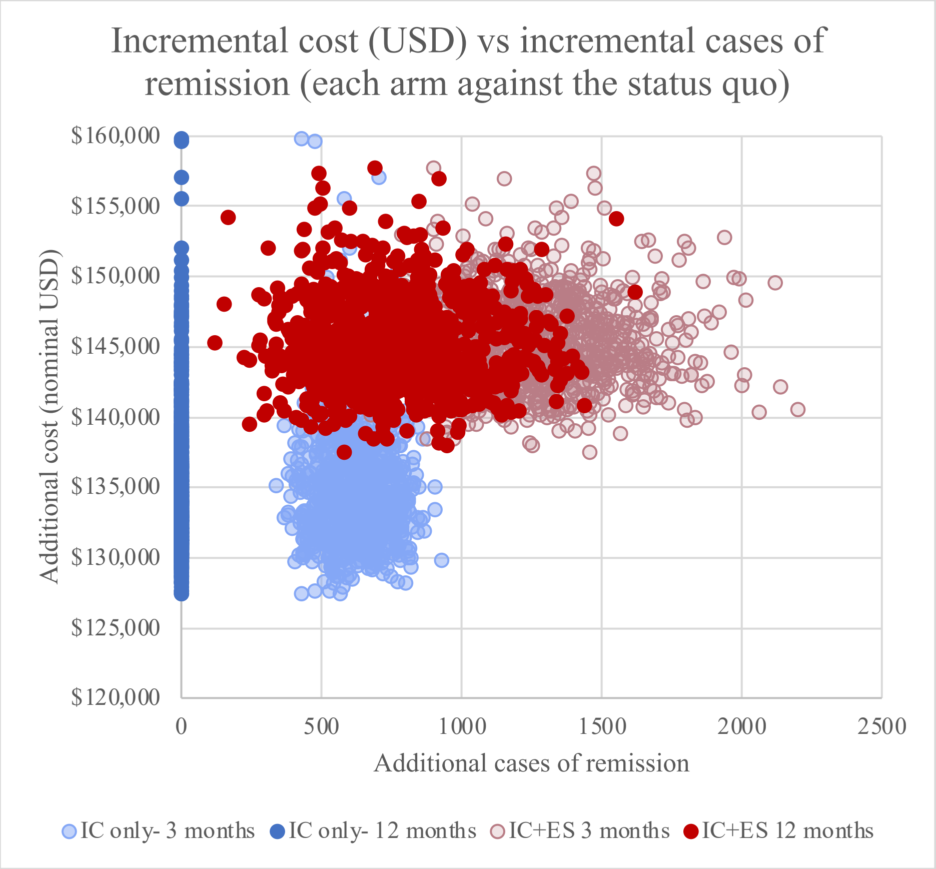

Incremental cost (USD) vs incremental cases of remission (each arm against the status quo)
$160,000
$155,000
$150,000
$145,000
$140,000
$135,000
$130,000
$125,000
$120,000
Additional cost (nominal USD)
0
500
1000
1500
2000
2500
Additional cases of remission
IC only- 3 months
IC only- 12 months
IC+ES 3 months
IC+ES 12 months

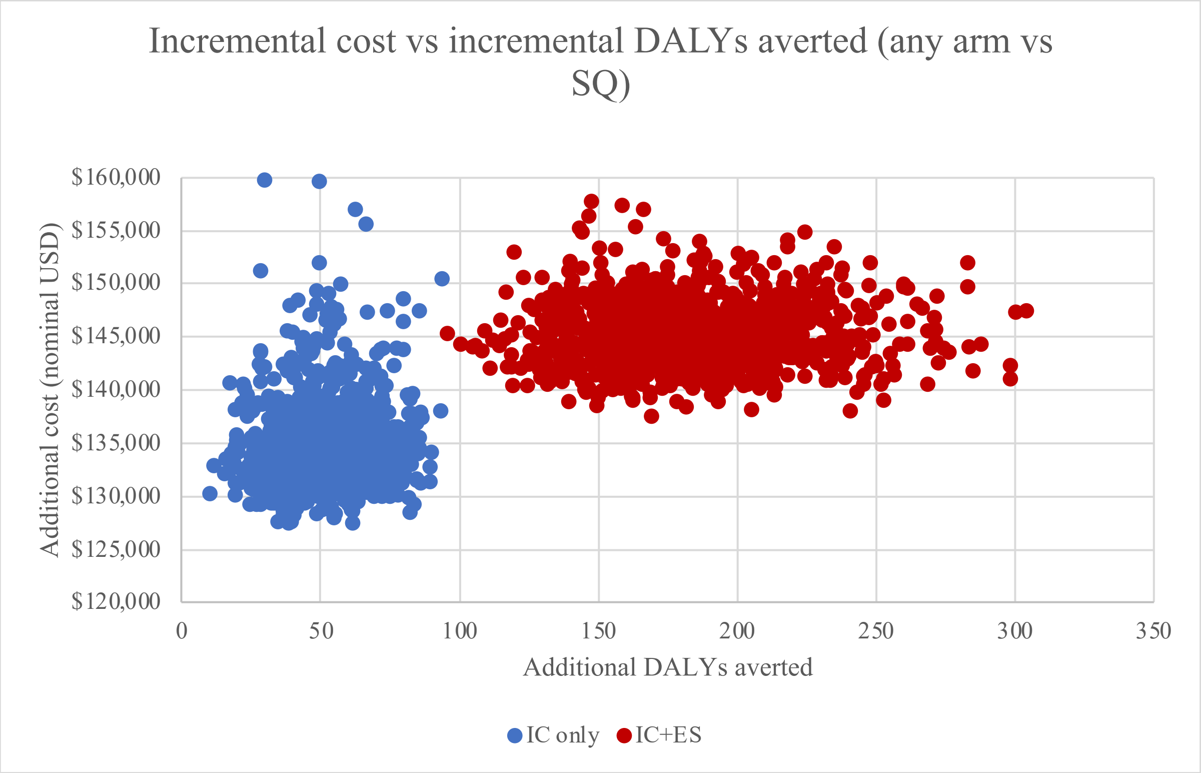

Incremental cost vs incremental DALYs averted (any arm vs SQ)
$160,000
$155,000
$150,000
$145,000
$140,000
$135,000
$130,000
$125,000
$120,000
Additional cost (nominal USD)
0
50
100
150
200
250
300
350
Additional DALYs averted
IC only
IC+ES

Year 2

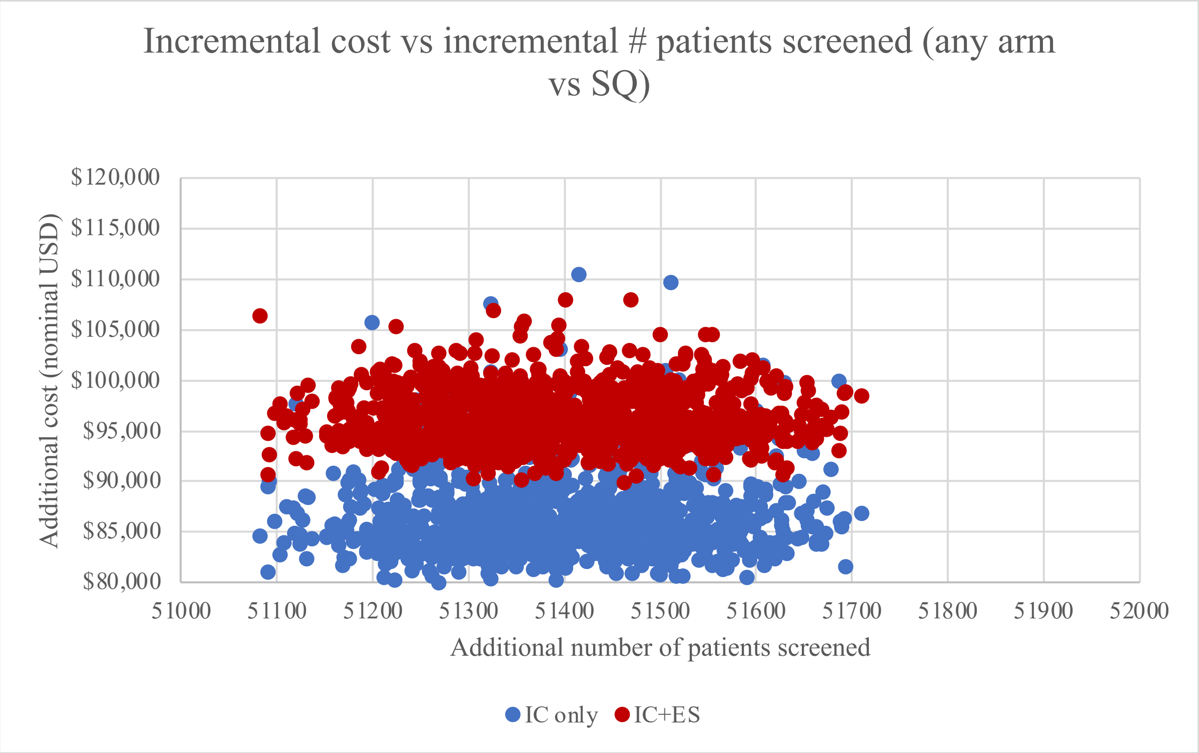

Incremental cost vs incremental # patients screened (any arm vs SQ)
Additional cost (nominal USD)
$120,000
$115,000
$110,000
$105,000
$100,000
$95,000
$90,000
$85,000
$80,000
51000
51100
51200
51300
51400
51500
51600
51700
51800
51900
52000
Additional number of patients screened
IC only
IC+ES

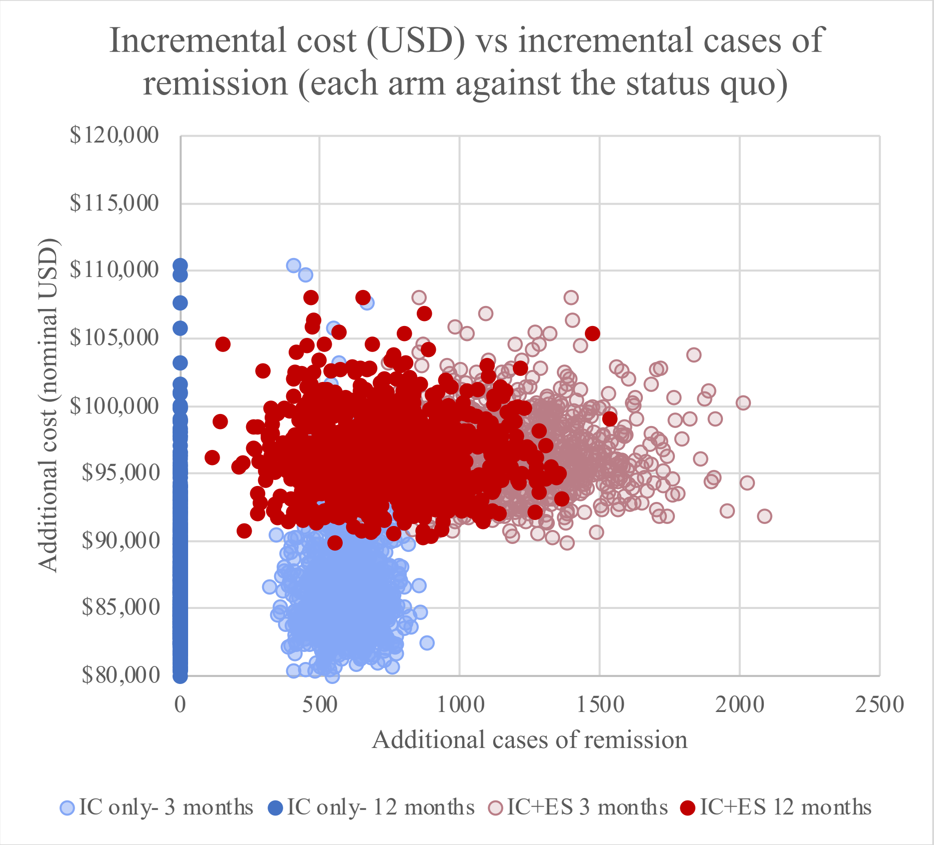

Incremental cost (USD) vs incremental cases of remission (each arm against the status quo)
Additional cost (nominal USD)
$120,000
$115,000
$110,000
$105,000
$100,000
$95,000
$90,000
$85,000
$80,000
0
500
1000
1500
2000
2500
Additional cases of remission
IC only- 3 months
IC only- 12 months
IC+ES 3 months
IC+ES 12 months

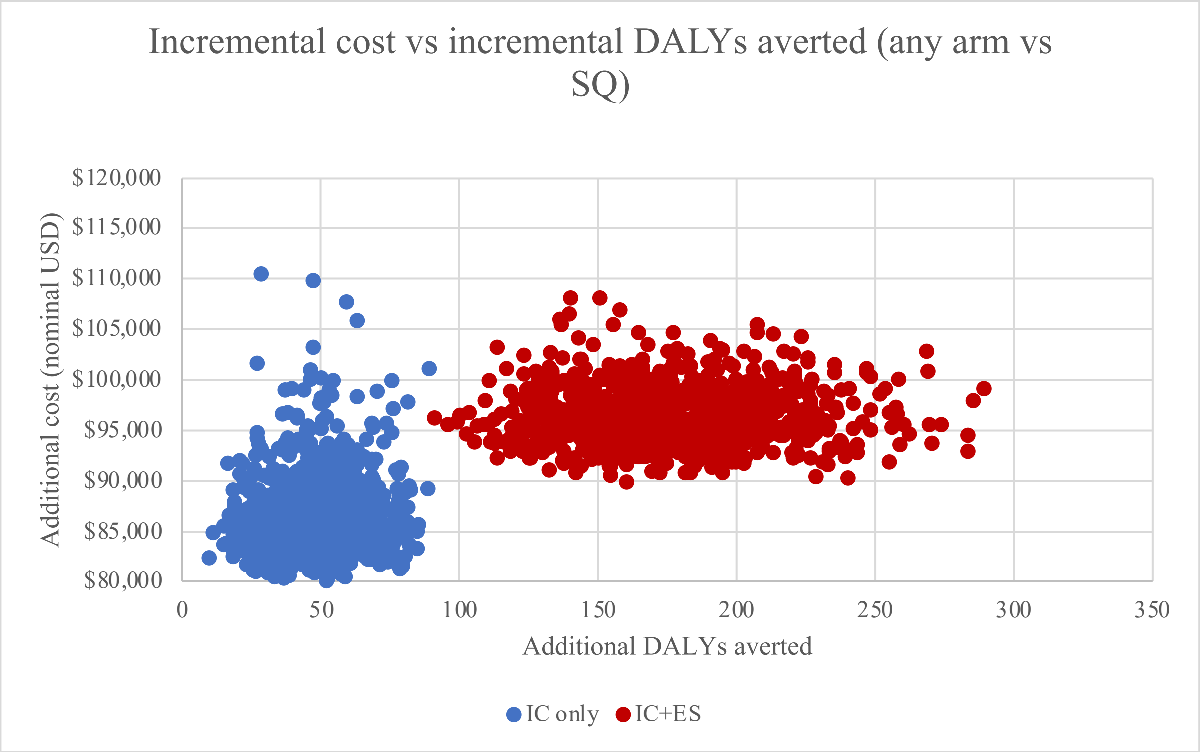

Abbreviations: US dollar (USD); Disability-Adjusted Life Year (DALY); Status quo (SQ); Internal Champion (IC); External Supervision (ES). Each point shows the change in effectiveness (e.g., incremental number of remissions at 3 months) and the incremental costs for one of the 1000 simulated trials in the basic implementation package (IC only) or the enhanced package including external supervision (IC+ES) versus the status quo.

Appendix 6. Cost-effectiveness frontiers.

We present cost-effectiveness frontiers for each of the outcomes of interest. Cost-effectiveness frontiers (CEFs) allow to simultaneously compare the three alternatives (IC+ES, IC-only, and SQ) to each other. They show the probability that each of the available alternatives has the highest net monetary benefit at different WTPTs.

Net monetary benefits (NMB) are calculated in the following way:

Willingness to pay threshold for a given outcome (WTPT) *x* Quantity of such outcome – Incremental Cost

A positive net monetary benefit indicates that a certain option is cost effective relative to its comparator.

For example, in a scenario in which the WTPT for additional remission at 3 months is $200, 1,400 additional remissions are achieved, and incremental costs are $200,000, the NMB is:

- $200/remission * 1,400 remissions – $200,000 = $80,000. As this value is positive, this alternative would be considered cost effective under a WTPT of $200/remission.

The CEFs confirm the results of the CEACs. In terms of cost per additional remission achieved or DALY averted, the probability of IC+ES becoming the optimal strategy increases as the WTPT increases, while IC-only is never the preferred option. However, the opposite is true in relation to cost per patient screened (IC+ES is never the optimal strategy, but the likelihood that IC-only is the preferred option is higher for higher WTPTs).

Year 1

Cost-effectiveness frontier. Cost per additional patient screened. Year 1.

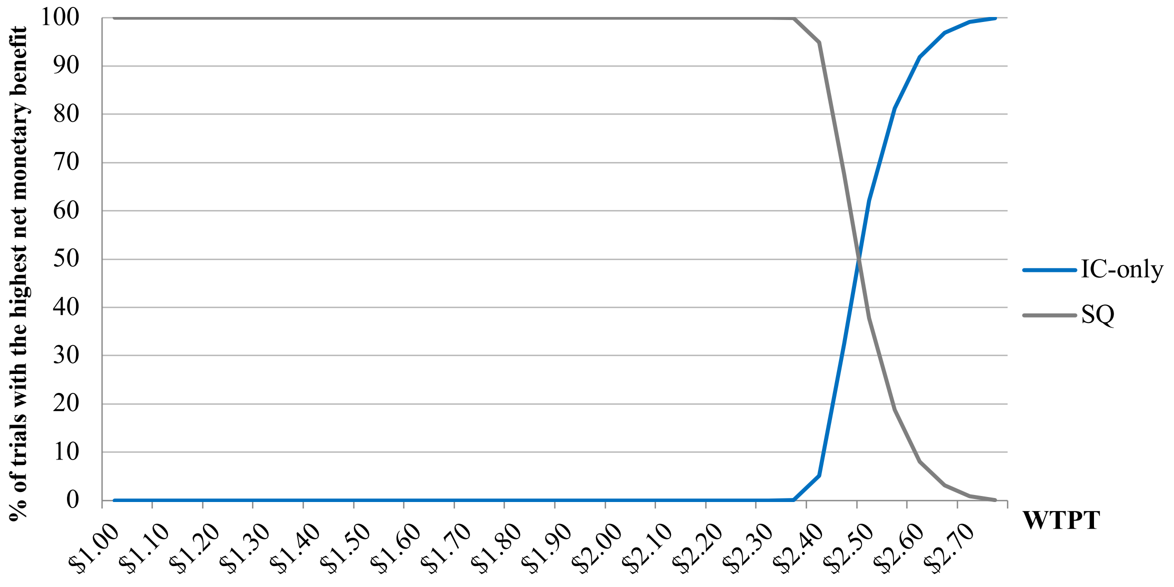

Cost-effectiveness frontier. Cost per additional remission at 3 months. Year 1.

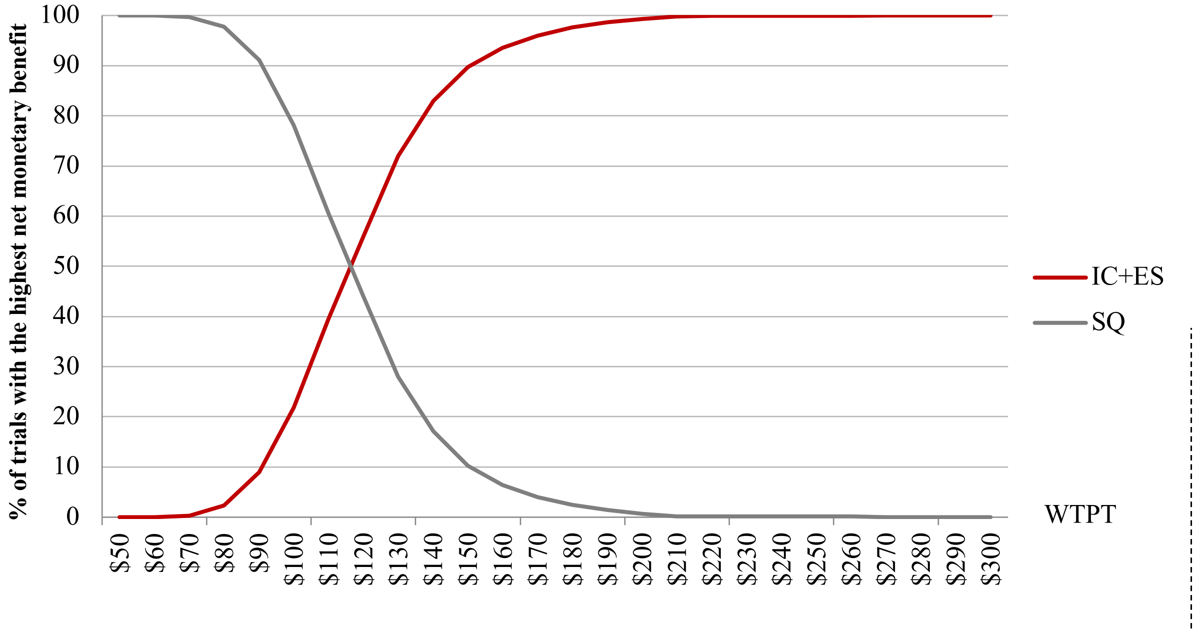

Cost-effectiveness frontier. Cost per additional remission at 12 months. Year 1.

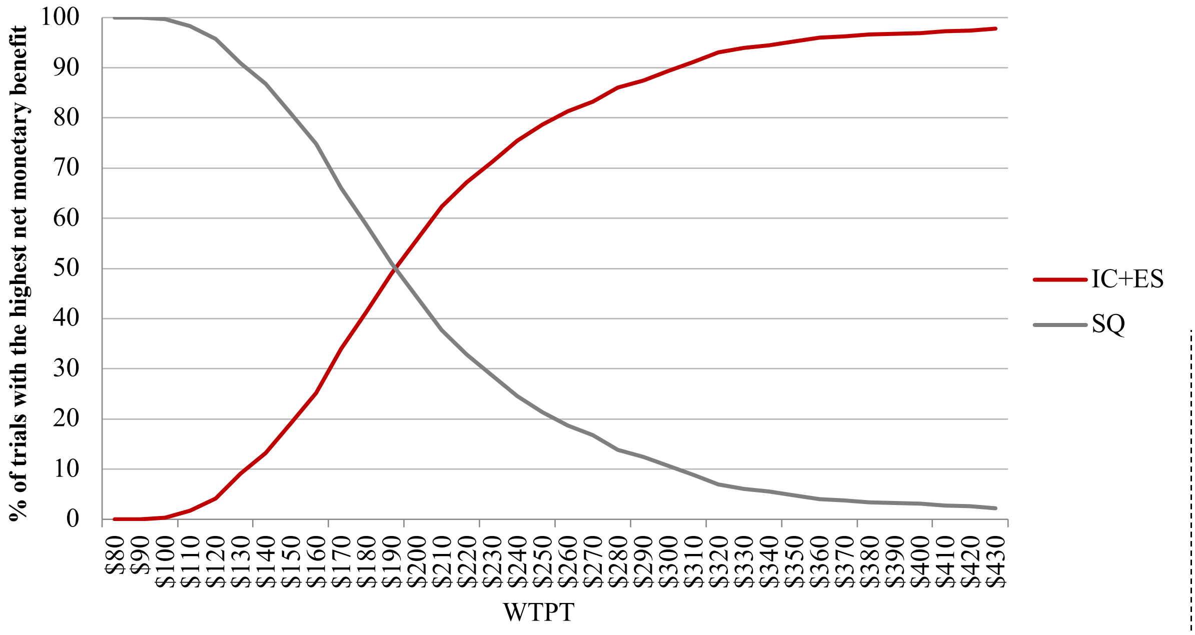

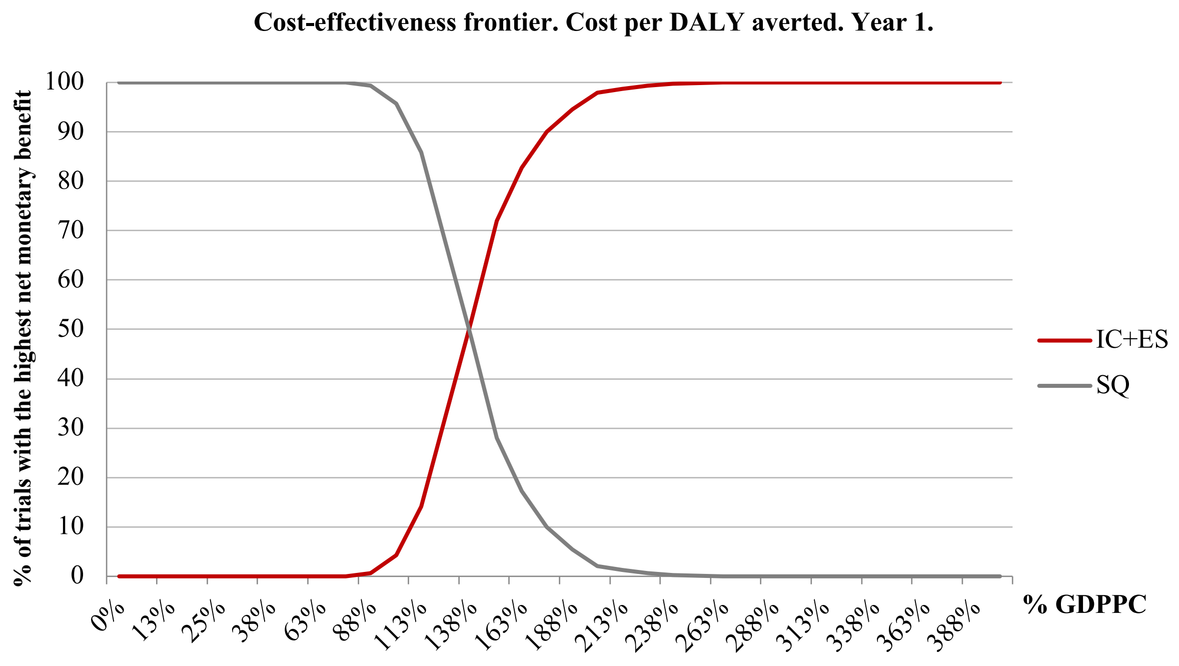

Cost-effectiveness frontier. Cost per DALY averted. Year 1.
% of trials with the highest net monetary benefit
0
10
20
30
40
50
60
70
80
90
100
0%
13%
25%
38%
63%
88%
113%
138%
163%
188%
213%
238%
263%
288%
313%
338%
363%
388%
% GDPPC
IC+ES
SQ

Year 2

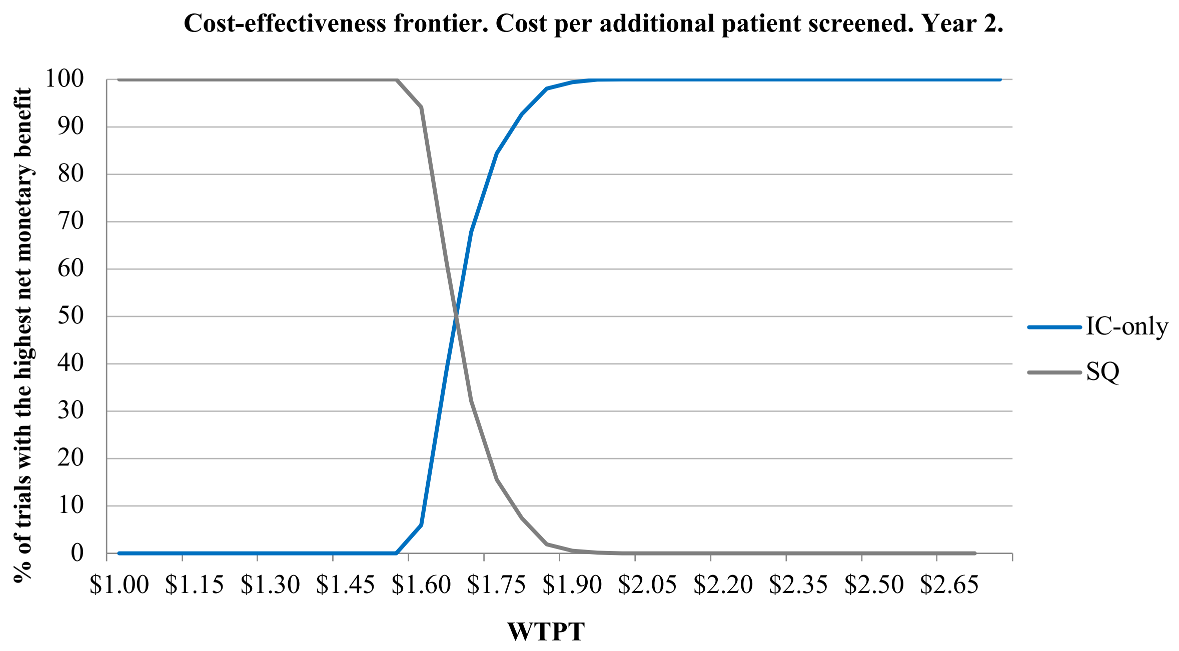

Cost-effectiveness frontier. Cost per additional patient screened. Year 2.
% of trials with the highest net monetary benefit
0
10
20
30
40
50
60
70
80
90
100
$1.00
$1.15
$1.30
$1.45
$1.60
$1.75
$1.90
$2.05
$2.20
$2.35
$2.50
$2.65
WTPT
IC-only
SQ

Cost-effectiveness frontier. Cost per additional remission at 3 months. Year 2.

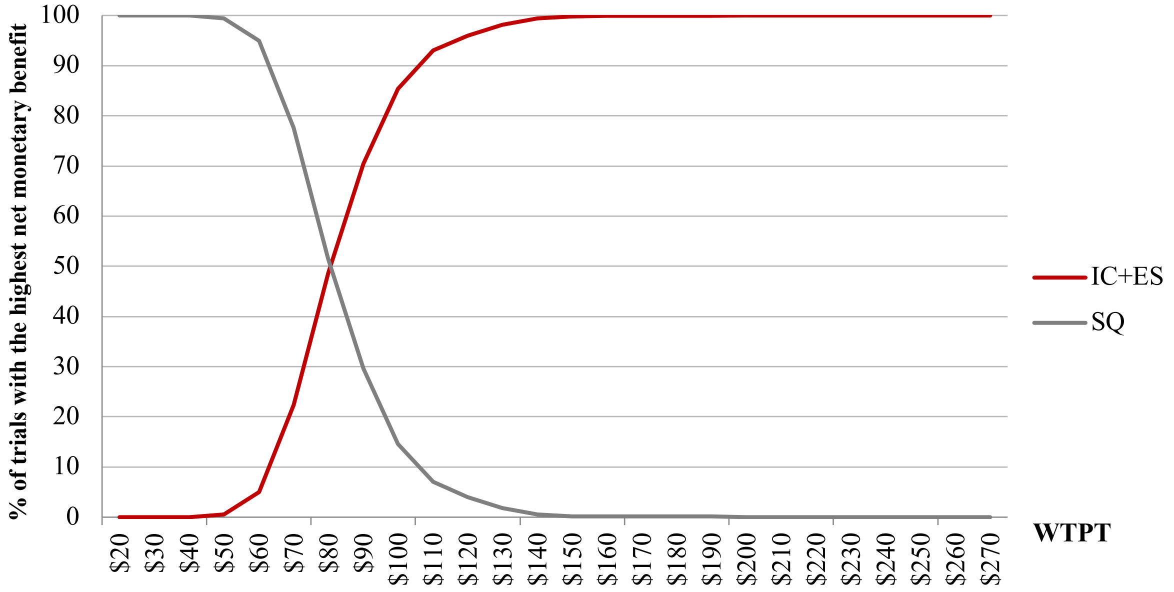

Cost-effectiveness frontier. Cost per additional remission at 12 months. Year 2.

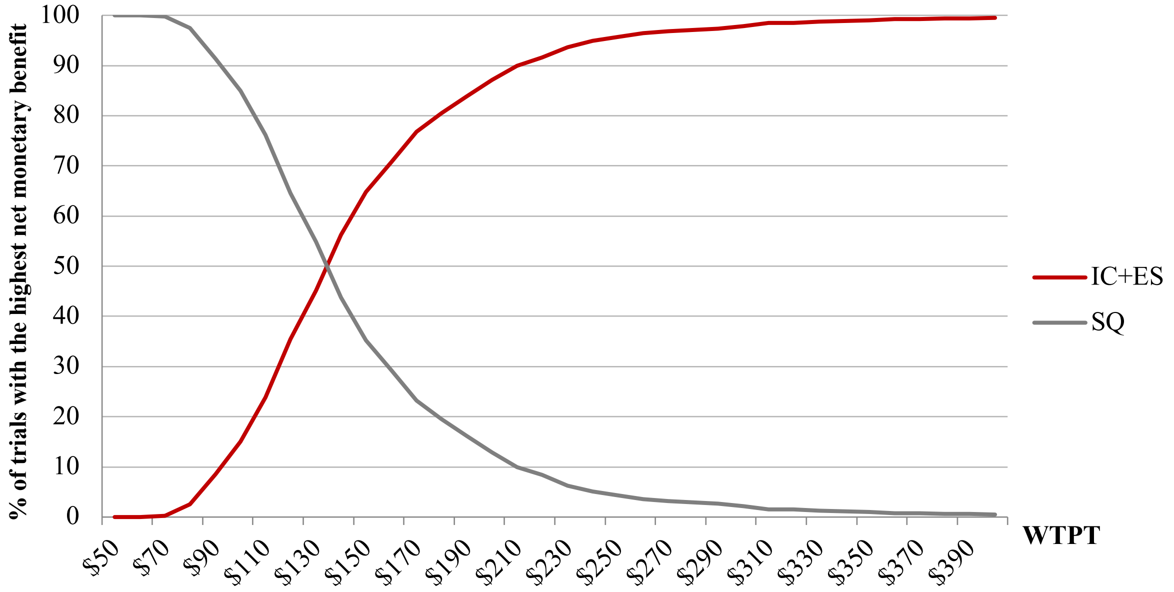

Cost-effectiveness frontier. Cost per DALY averted. Year 2.

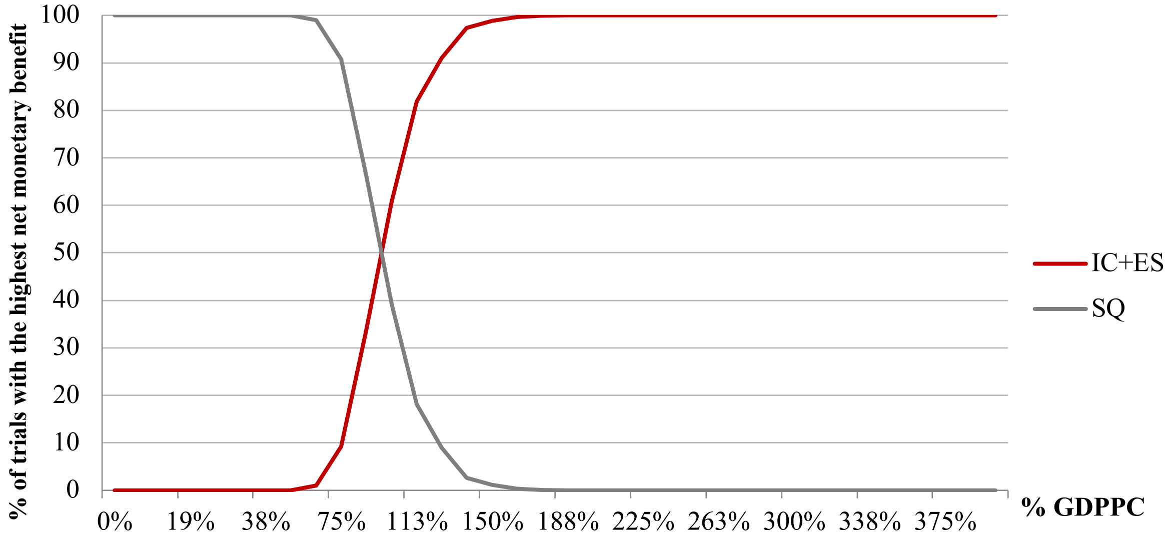

Appendix 7. Cost effectiveness of IC-only vs IC+ES.

Year 1

	Number of screened patients	*Expected number of patients with depression*	*Number of patients in remission at 3 months*	*Number of patients in remission at 12 months*	*Total depression related DALYs*	*Total cost (in million MWK)*	*Total cost (in nominal USD$)*
IC only	54,098 (53,887 – 54,322)	3,294 (3,214 – 3,366)	1,184 (1,073 – 1,302)	1,483 (1,354 – 1,612	538 (470 – 604)	98.5 (94.9 – 104.1)	134,867 (130,019 – 142,557)
IC+ES	54,098 (53,887 – 54,322)	3,294 (3,214 – 3,366)	1,828 (1,460 – 2,240)	2,255 (1,854 – 2,723)	396 (330 – 459)	106.0 (102.7 – 109.9)	145,255 (140,712 – 150,514)
	Additional # patients screened	Additional # patients in remission at 3 months	Additional # patients in remission at 12 months	Additional DALYs averted	Additional cost (in million MWK)	Additional cost (in USD$)	
IC only	Reference	Reference	Reference	Reference	Reference	Reference	
IC+ES	0	644 (311 – 1,014)	772 (423 – 1,199)	134 (90 – 185)	7.6 (1.0 – 12.7)	10,387 (1,349 – 17,365)	
	ICER per one additional patient screened (in nominal USD$)	ICER per one additional patient in remission at 3 months (in nominal USD$)	ICER per one additional patient in remission at 12 months (in nominal USD$)	ICER per one additional DALY averted (in nominal USD$)			
IC only	Reference	Reference	Reference	Reference			
IC+ES	Dominated	18 (2– 40)	15 (2 – 32)	81 (11 – 154)			

Year 2

	ICER per one additional patient screened (in nominal USD$)	*ICER per one additional patient in remission at 3 months (in nominal USD$)*	*ICER per one additional patient in remission at 12 months (in nominal USD$)*	*ICER per one additional DALY averted (in nominal USD$)*	*Total discounted cost (in million MWK)*	*Total discounted cost (in nominal USD$)*
IC only	Reference	Reference	Reference	Reference	Reference	Reference
IC+ES	Dominated	18 (2– 40)	15 (2 – 32)	81 (11 – 154)	7.2(0.9 – 12.0)	9,868 (1,282 – 16,497)

The figures below represent the Cost-Effectiveness Acceptability Curves (CEACs) for both the primary (IC vs status quo and IC+ES versus status quo) and secondary (IC-only vs IC+ES) analyses. These are shown in the left and right hand-side panels, respectively.

Year 1

a. Incremental cost per additional case of remission.

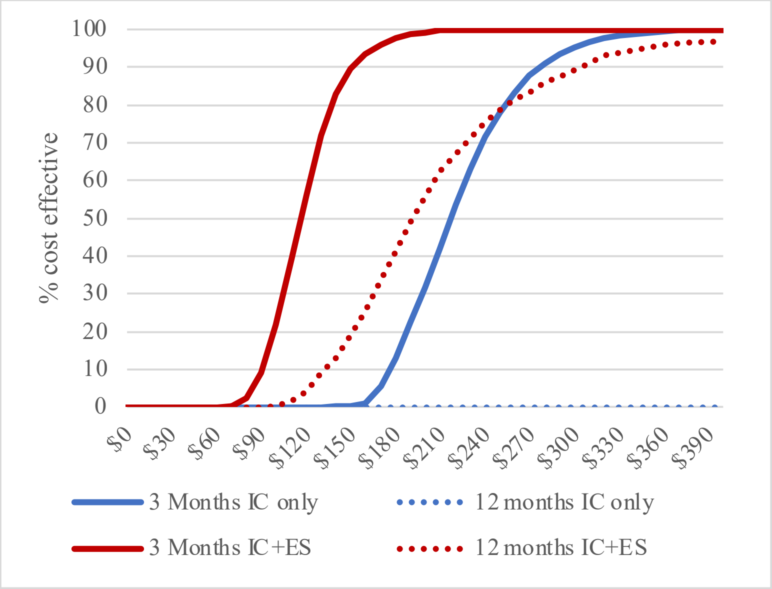

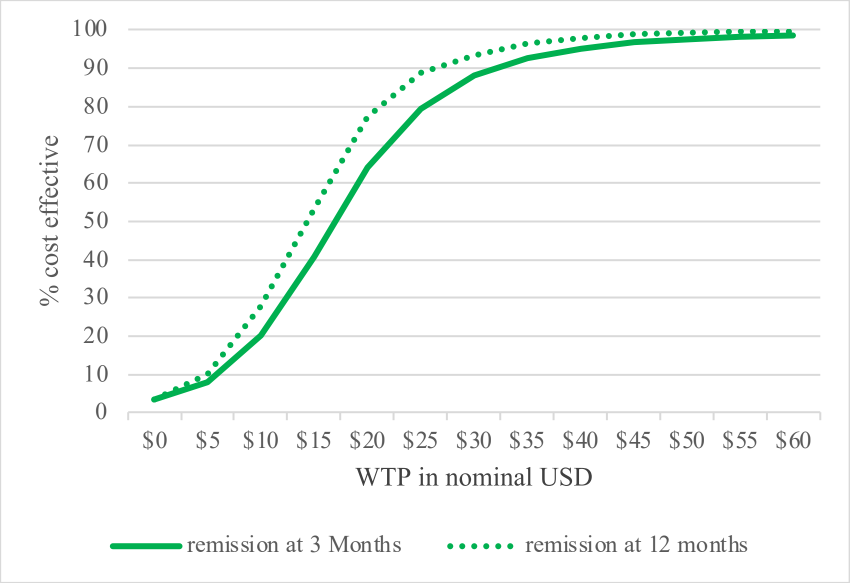

b. Incremental cost per DALY averted.

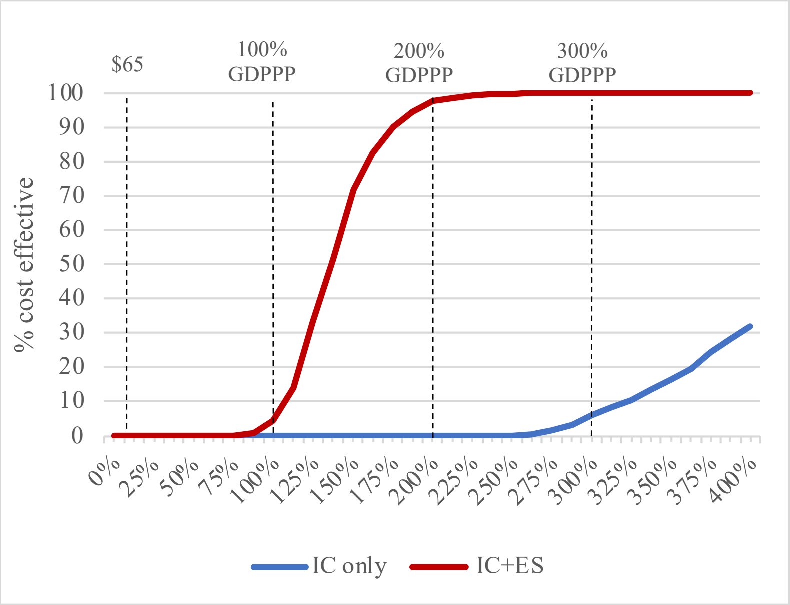

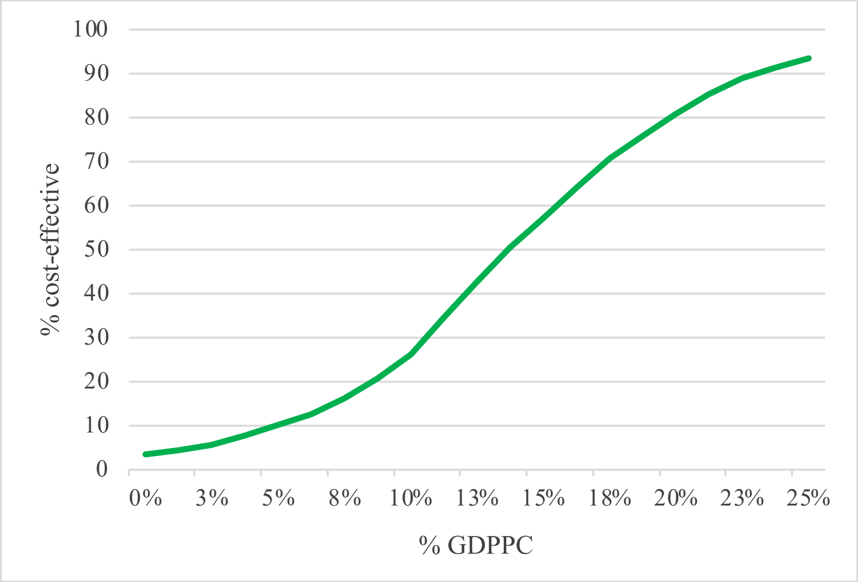

Abbreviations: Gross Domestic Product Per Capita (GDPPC); Willingness to Pay Threshold (WTPT); Internal Champion (IC); External Supervision (ES); Disability-Adjusted Life Year (DALY); Cost-Effectiveness Acceptability Curves (CEACs). The CEACs on the right-hand side illustrate the percentage of simulations in which the IC+ES alternative is cost-effective compared to the IC+only one for different WTPTs. In other words, when the IC-only is the standard of care, IC+ES becomes cost-effective at least 90% of the time at WTPTs $30 and $25, and 23% of GDPPC (approximately $130) for additional remission at 3 months, remission at 12 months, and DALY averted, respectively. By contrast, the CEACs on the right-hand side compare the cost effectiveness of each IC-only and IC+ES vs a status quo of no depression treatment.

Year 2

a. Incremental cost per additional case of remission.

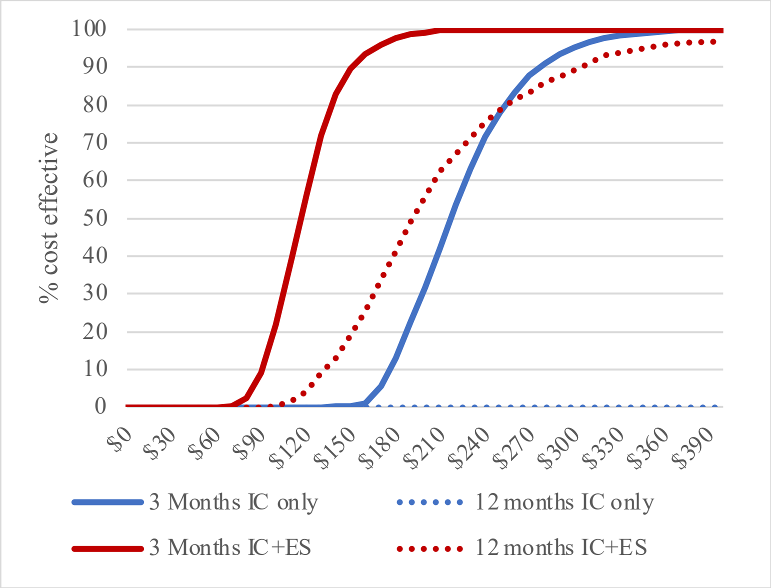

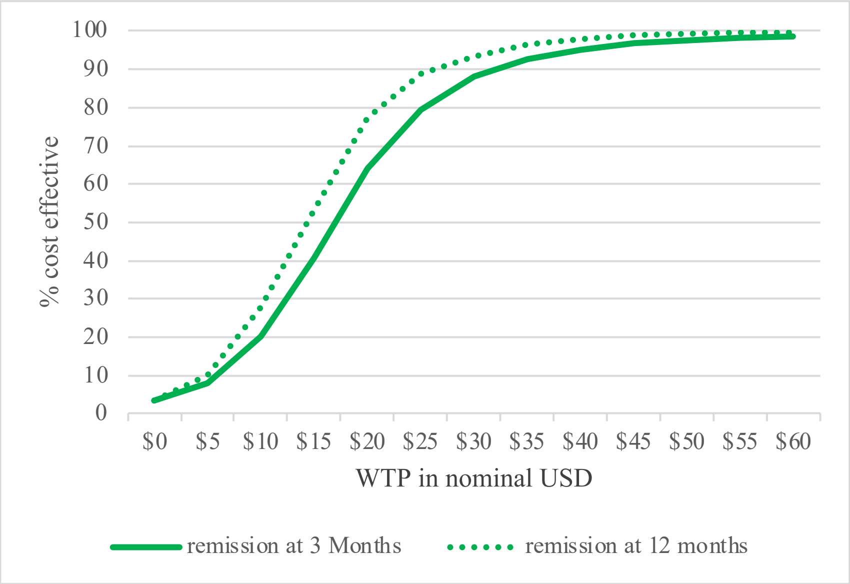

b. Incremental cost per DALY averted.

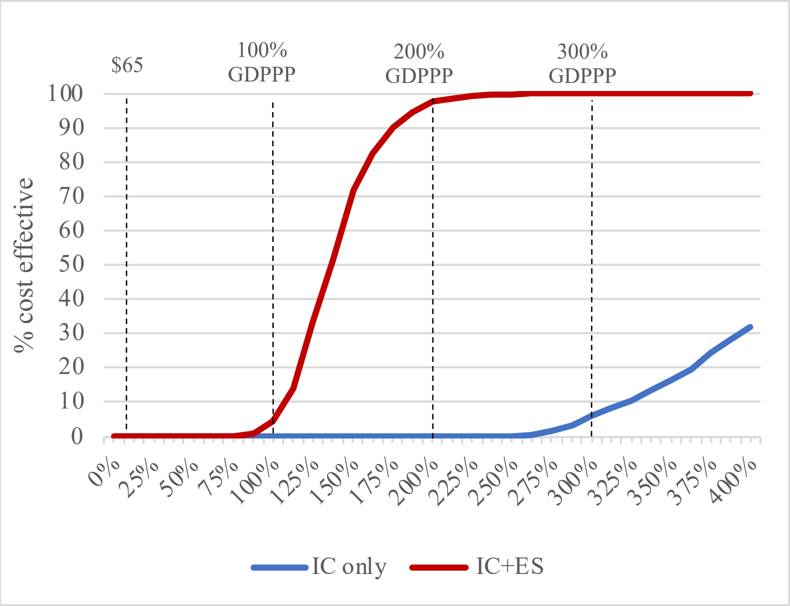

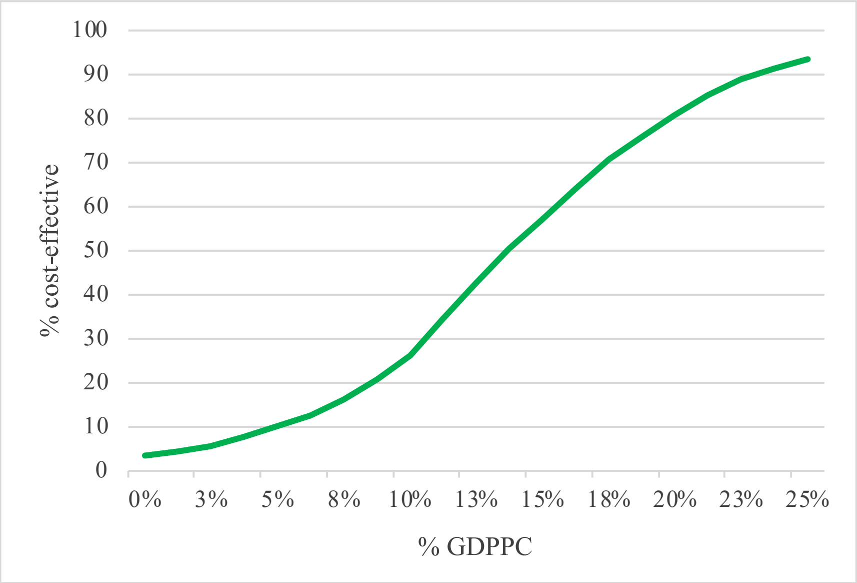

Abbreviations: Gross Domestic Product Per Capita (GDPPC); Willingness to Pay Threshold (WTPT); Internal Champion (IC); External Supervision (ES); Disability-Adjusted Life Year (DALY); Cost-Effectiveness Acceptability Curves (CEACs). The CEACs on the right-hand side illustrate the percentage of simulations in which the IC+ES alternative is cost-effective compared to the IC+only one for different WTPTs. In other words, when the IC-only is the standard of care, IC+ES becomes cost-effective at least 90% of the time at WTPTs $30 and $25, and 23% of GDPPC (approximately $130) for additional remission at 3 months, remission at 12 months, and DALY averted, respectively. By contrast, the CEACs on the right-hand side compare the cost effectiveness of each IC-only and IC+ES vs a status quo of no depression treatment.
