# Supplementary material for: Implementation strategies to build mental health-care capacity in Malawi: a health-economic evaluation
Source: Lancet Glob Health. Author manuscript; Available in PMC 2024 Apr 1. (PMC10958395; doi:10.1016/S2214-109X(23)00597-1)
Supplement: 2 [file NIHMS1976842-supplement-2.pdf]

THE LANCET
Global Health

Supplementary appendix 2

This Equitable Partnership Declaration (EPD) was submitted by the authors, and we reproduce it as supplied. It has not been peer reviewed. *The Lancet*'s editorial processes have not been applied to the EPD.

Supplement to: Yanguela J, Pence BW, Udedi M, et al. Implementation strategies to build mental health-care capacity in Malawi: a health-economic evaluation. *Lancet Glob Health* 2024; published online Feb 23. https://doi.org/10.1016/S2214-109X(23)00597-1.

Equitable Partnership Declaration questions

Researcher considerations

1. Please detail the involvement that researchers who are based in the region(s) of study had during a) study design; b) clinical study processes, such as processing blood samples, prescribing medication, or patient recruitment; c) data interpretation; and d) manuscript preparation, commenting on all aspects. If they were not involved in any of these aspects, please explain why.

   This question is intended for international partnerships; if all your authors are based in the area of study, this question is not applicable.

   This should include a thorough description of their leadership role(s) in the study. Are local researchers named in the author list or the acknowledgements, or are they not mentioned at all (and, if not, why)? Please also describe the involvement of early career researchers based in the location of the study. Some of this information might be repeated from the Contributors section in the manuscript. Note: we adhere to ICMJE authorship criteria when deciding who should be named on a paper.

**a) Study design:**<br>Colleagues from the University of North Carolina in the US, UNC Project Malawi, the Malawi Ministry of Health, and the Kamuzu University of Health Sciences in Malawi collaborated in the design of the parent trial (specifically in defining the evidence-based intervention to be deployed and the implementation strategies to be tested that were relevant to the Malawian health system context), the creation of a prospective cost and time-and motion data collection strategy, the development of the structure of the decision analytical model, and the compilation of data inputs for the cost-effectiveness analysis.
**b) Clinical study processes:**<br>Colleagues from the University of North Carolina in the US, UNC Project Malawi, the Malawi Ministry of Health, and the Kamuzu University of Health Sciences in Malawi collaborated in defining clinical study processes for the parent trial (specifically in developing depression treatment protocols that were relevant to the Malawian clinical and health system context) and operationalizing these clinical processes into the structure of the decision-analytical model.
**c) Data interpretation:**<br>Colleagues from the University of North Carolina in the US, UNC Project Malawi, the Malawi Ministry of Health, and the Kamuzu University of Health Sciences in Malawi collaborated in data interpretation, specifically in discussing analysis results and developing and delivering presentations of key study findings.
**d) Manuscript preparation:**<br>Colleagues from the University of North Carolina in the US, UNC Project Malawi, the Malawi Ministry of Health, and the Kamuzu University of Health Sciences in Malawi collaborated in manuscript preparation. Specifically, the first author drafted the manuscript and all co-authors

reviewed and edited the manuscript for key intellectual content, in particular elaborating on presentation of context and on policy implications and recommendations.

2. Were the data used in your study collected by authors named on the paper, or have they been extracted from a source such as a national survey? ie, is this a secondary analysis of data that were not collected by the authors of this paper. If the authors of this paper were not involved in data collection, how were data interpreted with sufficient contextual knowledge?

   The Lancet Global Health *believe contextual understanding is crucial for informed data analysis and interpretation.*

   The data used in this study were collected by authors named on the paper.

3. How was funding used to remunerate and enhance the skills of researchers and institutions based in the area(s) of study? And how was funding used to improve research infrastructure in the area of study?

   Potentially effective investments into long-term skills and opportunities within institutions could include training or mentorship in analytical techniques and manuscript writing, opportunities to lead all or specific aspects of the study, financial remuneration rather than requiring volunteers, and other professional development and educational opportunities.

   Improvements to research infrastructure could be funding of extended trial designs (such as platform trials) and use of master protocols to enable these designs, establishment of long-term contracts for research staff, building research facilities, and local control of funding allocation.

   Skills:
   The SHARP Study (i.e., the parent trial from which cost and effectiveness data were obtained) included an explicit capacity building core that engaged in a range of capacity building activities over the course of the five-year grant period. These activities included (1) short courses in implementation science and global mental health for Malawian and Tanzanian researchers, NGO staff, and policy makers and (2) pilot grant awards to Malawian and Tanzanian teams involving one researcher or NGO staff and one policy maker to conduct small research projects focusing on mental health and implementation science. Additionally, the named Malawian co-authors all received remuneration from the study and assumed important leadership roles in study implementation, building their skills in research leadership.

   Research infrastructure:
   The activities listed above increased research infrastructure by providing experience in the delivery of small pilot grants and in management of the trial itself.

4. How did you safeguard the researchers who implemented the study?

Please describe how you guaranteed safe working conditions for study staff, including provision of appropriate personal protective equipment, protection from violence, and prevention of overworking.

For the SHARP Study, research staff were employed by UNC Project-Malawi, which has well developed operational policies regarding safe working conditions and hours worked. At the onset of the COVID-19 pandemic, all study staff initially left their research sites and continued follow-up interviews by phone. Subsequently, UNC Project-Malawi developed and implemented protocols around provision and use of personal protective equipment to resume face-to-face data collection.

Benefits to the communities and regions of study

5. How does the study address the research and policy priorities of its location?

How were the local priorities determined and then used to inform the research question? Who decided which priorities to take forward? Which elements of the study address those priorities?

Colleagues from the University of North Carolina in the US, the Malawi Ministry of Health, and the Kamuzu University of Health Sciences in Malawi collaborated in the design of the parent trial (specifically in defining the evidence-based intervention to be deployed and the implementation strategies to be tested that were relevant to the Malawian health system context), the creation of a prospective cost and time-and motion data collection strategy, the development of the structure of the decision analytical model (including relevant simplifying assumptions and comparators to assess), and in the compilation of necessary data inputs.

These colleagues also jointly decided which outcomes to assess as part of the cost effectiveness analysis (including cost per patient screened, remission achieved and DALY averted), and how to interpret the data in light of Malawi's specific socio-demographic profile.

6. How will research products be shared in the community of study?

For instance, will you be providing written or oral layperson summaries for non-academic information sharing? Will study data be made available to institutions in the region(s) of study? The Lancet Global Health *encourages authors to translate the summary (abstract) into relevant languages after paper editing; do you intend to translate your summary?*

Findings from cost-effectiveness analysis have already been disseminated to key parties in the Malawi Ministry of Health.

Other analyses stemming from the parent trial have also been disseminated to both the Ministry and participating clinics and have led to policy changes. Furthermore, efficacy data from the parent trial are publicly available to all researchers on the NIMH Data Archive.

English is the official language of Malawi for medical, scientific, and policy documents and is therefore an appropriate language for this manuscript.

7. How were individuals, communities, and environments protected from harm?

a) *How did you ensure that sensitive patient data was handled safely and respectfully? Was there any potential for stigma or discrimination against participants arising from any of the procedures or outcomes of the study?*

In the parent trial, informed consent, enrolment, and data collection activities occurred in a private location. Participant data were coded with a non-identifying ID number, stored securely in either locked locations or on secure servers, and stored separately from participant names and the name-ID link log. Procedures and outcomes of the study did not pose a risk for stigma or discrimination.<br><br>Only aggregate level data from the parent trial was used to inform the cost-effectiveness analysis.

b) *Might any of the tests be experienced as invasive or culturally insensitive?*

In the parent trial, participant involvement was limited to answering questionnaires and measuring height, weight, and blood pressure following the procedures routinely used in clinical care.

c) *How did you determine that work was sensitive to traditions, restrictions, and considerations of all cultural and religious groups in the study population?*

The development of study protocols in the parent trial was led by Malawian team members with extensive experience with the population being recruited.

d) *Were biowaste and radioactive waste disposed of in accordance with local laws?*

NA

e) *Were any structures built that would have impacted members of the community or the environment (such as handwashing facilities in a public space)? If so, how did you ensure that you had appropriate community buy-in?*

NA

f) *How might the study have impacted existing health-care resources (such as staff workloads, use of equipment that is typically employed elsewhere, or reallocation of public funds)?*

As an implementation study, the purpose of the parent trial was to test implementation strategies to support the integration of a new clinical practice – integrated depression screening and treatment – into existing care using existing staff. The Malawi Ministry of Health was closely involved in stating this integration as a Ministry priority, communicating with facilities about the integration, and leading the implementation strategies to aide facilities in problem-solving about the best and most sustainable ways of integrating the new practice into existing care.

8. Finally, please provide the title (eg, Dr/Prof, Mr/Mrs/Ms/Mx), name, and email address of an author who can be contacted about this statement. This can be the corresponding author.

Name: Brian Pence, senior author
Email: bpence@unc.edu
